# Accurate label-free reaction kinetics determination using initial rate heat measurements

**DOI:** 10.1038/srep16380

**Published:** 2015-11-17

**Authors:** Kourosh Honarmand Ebrahimi, Peter-Leon Hagedoorn, Denise Jacobs, Wilfred R. Hagen

**Affiliations:** 1 Department of Biotechnology, Delft University of Technology, Julianalaan 67, 2628 BC Delft, The Netherlands; 2 DSM Biotechnology Center, Alexander Fleminglaan 1, 2613 AX Delft, the Netherlands

## Abstract

Accurate label-free methods or assays to obtain the initial reaction rates have significant importance in fundamental studies of enzymes and in application-oriented high throughput screening of enzyme activity. Here we introduce a label-free approach for obtaining initial rates of enzyme activity from heat measurements, which we name initial rate calorimetry (IrCal). This approach is based on our new finding that the data recorded by isothermal titration calorimetry for the early stages of a reaction, which have been widely ignored, are correlated to the initial rates. Application of the IrCal approach to various enzymes led to accurate enzyme kinetics parameters as compared to spectroscopic methods and enabled enzyme kinetic studies with natural substrate, e.g. proteases with protein substrates. Because heat is a label-free property of almost all reactions, the IrCal approach holds promise in fundamental studies of various enzymes and in use of calorimetry for high throughput screening of enzyme activity.

The ability to measure the initial reaction rates is of fundamental importance for understanding the kinetics of various chemical and biological reactions such as conversion of substrates to products by heterogeneous or homogenous catalysts, or by enzymes, and protein folding. Different techniques have been developed to measure the initial rate of each of these processes in order to obtain detailed information regarding their kinetics. For example circular dichroism and optical tweezers for studying protein folding^1^,^2^,^3^,^4^,^5^, or colorimetric assays based on UV-visible or fluorescence spectroscopy for studying enzyme-catalyzed reactions^6^,^7^,^8^,^9^,^10^,^11^,^12^. Unfortunately, because the natural substrates/products of many enzymes neither have a color nor are fluorogenic, the application of colorimetric assays for initial rate measurements of enzymes with their natural substrates, which is essential in fundamental studies of enzymes and is desirable in high throughput screening of enzyme inhibitors as possible drug candidates, is very limited. For example the initial rate activity of enzymes such as kinases, phosphatases, proteases, or sulfatases, which are important drug targets^13^,^14^,^15^,^16^, cannot be studied using natural substrates. To overcome limitations of the colorimetric assays various label-free methods for fast measurement of activity of enzymes have been developed such as DNA-based detection systems using fluorogenic probes^17^,^18^,^19^,^20^, blockage of supramolecular pores with substrates or products and subsequent fluorometric detection^21^, continues enzyme assays based on macrocycle-fluorescent dye complexes^22^, or monitoring enzyme activity using mesoporous silicone double layer^23^. A label-free property of all reactions is heat, which can be measured quantitatively using calorimetric techniques. Although methods based on the simulation of the complete calorimetric curves, which typically take minutes to hours to record, to obtain kinetics data have been developed^24^, there is no method available to obtain initial reaction rates from the heat measurements using calorimetry.

Here we present a new label-free approach, initial rate calorimetry (IrCal), for obtaining the initial reaction rates using isothermal titration calorimetry (ITC) (Fig. 1), which is a very sensitive calorimetric method and has been widely applied in chemistry and biology for measuring thermodynamic parameters of a ligand binding to a macromolecule^24^,^25^,^26^,^27^,^28^,^29^. Figure 1a shows the main elements of a common isothermal titration calorimeter and explains the basic principles of its operation, and Fig. 1b,c show the power recorded by an ITC instrument for an endothermic and an exothermic reaction respectively. We show that the IrCal approach can be used to accurately obtain enzyme kinetic parameters with natural substrates, to study inhibitors of enzymes, to test substrate specificity of an enzyme, and to measure activity of proteases with natural protein substrates.

## Results

### Recorded power is not a direct measure of the rate of enzymatic heat production

**Figure 2:**
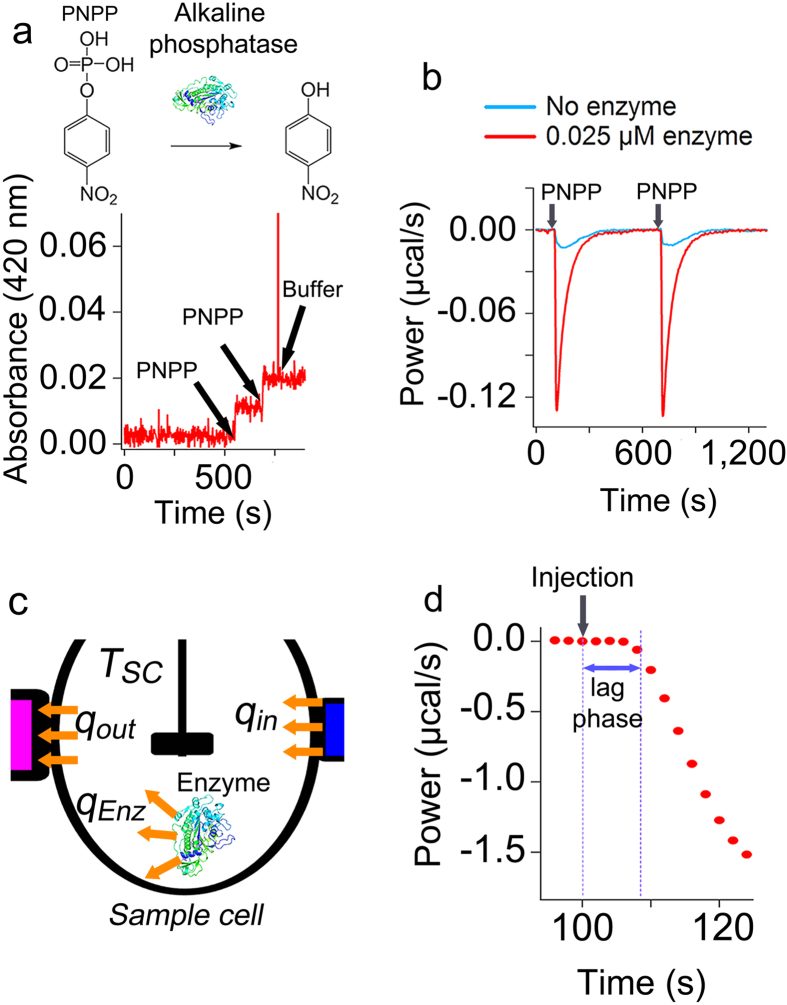
The heat flow rate measured by ITC is not a direct measure of the rate of heat generated or consumed by an enzymatic reaction. (**a**) Conversion of PNPP (0.5 μM) by alkaline phosphatase (25 nM) was measured by UV-visible spectroscopy, and (**b**) by ITC at 30 °C. (**c**) A schematic presentation of the heat transfer processes associated with an exothermic enzymatic reaction in the sample cell. The heat generated by the enzymatic reaction, increases the temperature inside the sample cell to T_SC_. The difference between the temperature inside the sample cell and its outer surface leads to heat flow rate towards the thermocouple (q_out_), which is translated by the PID controller to the heat flow rate that is needed to be applied by the feedback heater (q_in_). (**d**) A lag phase was observed in the measurement of heat generated by conversion of PNPP by alkaline phosphatase. The data show conversion of 4.3 μM PNPP by 5 nM alkaline phosphatase at 30 °C. The power recorded by ITC (**b**, and **d**) for conversion of PNPP by alkaline phosphatase is corrected for the power recorded due to heat of dilution of PNPP in the absence of enzyme.

Recently it has been assumed that the power recorded by modern ITC instruments is a direct measure of the rate of heat generated by an enzymatic reaction and based on this assumption different methods for simulation of ITC curves to obtain enzyme kinetic parameters were proposed^30^,^31^,^32^,^33^. To check whether this assumption is correct, for an enzyme-catalyzed reaction we compared online measurements using UV-visible spectroscopy with measurements using ITC. We recorded conversion of p-nitrophenyl phosphate (PNPP) by alkaline phosphatase using UV-visible spectroscopy (Fig. 2a) and ITC (Fig. 2b) under exactly the same conditions, i.e. the same substrate and enzyme concentrations and experimental conditions. It can be seen that while full conversion of a small amount (0.5 μM) of PNPP by alkaline phosphatase took less than 10 seconds (Fig. 2a), as measured by UV-visible spectroscopy, the power recorded for the enzymatic activity, i.e. after correction for the power recorded for the dilution of substrate in the absence of enzyme (Methods, and Supplementary Figure 1), took more than 300 seconds (Fig. 2b). This suggested that the response of ITC instrument to any heat effect in the sample cell (Fig. 2c) at any time during the measurement is behind the actual rate of heat generated by the enzyme. This delay is because of the thermal inertia of calorimeters and various time constants of the instrument^34^,^35^,^36^,^37^,^38^ such as the time constants of thermocouple and PID controller. Aa a result, the power measured by a calorimeter will never be directly equal to the actual rate of heat generated by an enzymatic reaction and the assumption that the recorded power by ITC is the same as the rate of heat generated by an enzyme is not correct. Furthermore, we observed that while the injection of substrate took 2–5 seconds, the recorded power after the inject of substrate remained almost flat for circa 8–12 seconds and only after this lag phase it changed (Fig. 2d). The lag phase (Methods) was circa 8 seconds for injection period of 2 seconds and it increased as the injection period increased (Supplementary Figure 1). This suggests that the lag phase is the time to reach full mixing at the stirring speed of 502 rpm, which we used (Methods). Because the lag phase is dependent on mixing time and is affected by instrumental related delays, it might slightly vary for different ITC instruments, it is readily determined graphically as explained in the Methods.

### The initial ITC data are linearly related to the initial rate of a reaction

**Figure 3:**
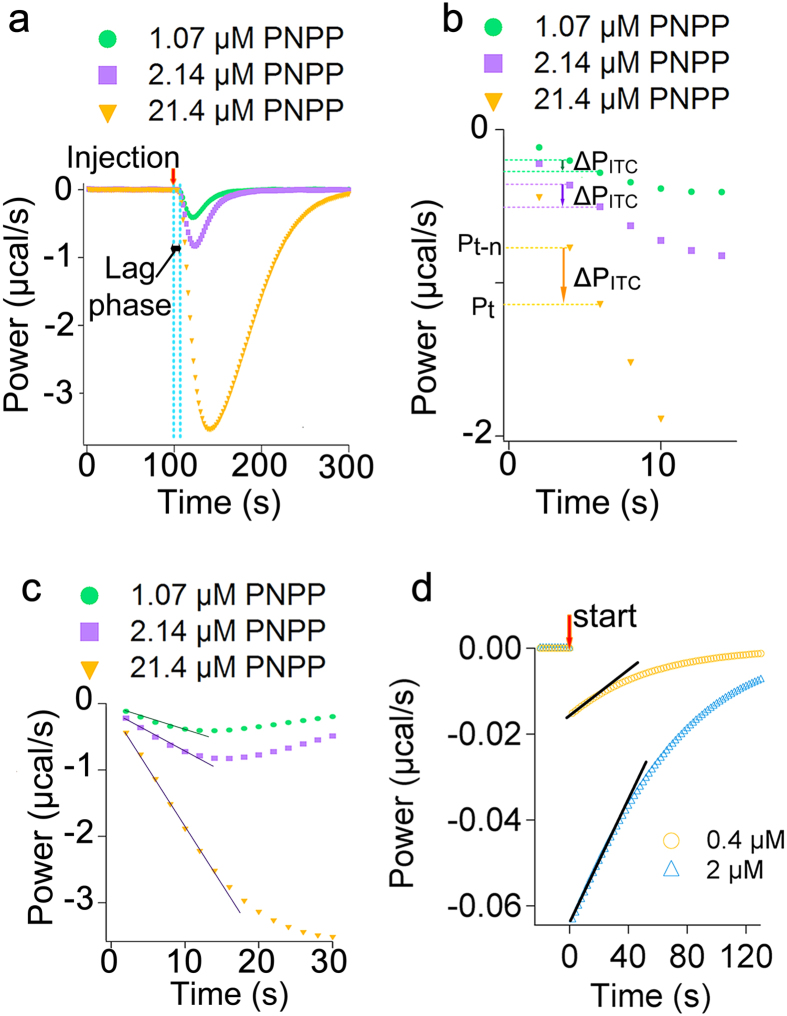
The early data points recorded by ITC are linearly related to the rate of enzymatic reaction. (**a**) The power change recorded for the exothermic conversion of different amounts of PNPP by alkaline phosphatase (5 nM). A few seconds after injection of substrate, i.e. after the lag phase, the heat production by enzymatic reaction is detected. (**b**) The first data point after the lag phase was considered as the first measurement point (t = 2 s) and all the other data were shifted in time accordingly. For the early data points after the lag phase the change in the power of two subsequent data points (ΔP_ITC_) increases as the rate of enzymatic reaction increases by addition of more substrate. (**c**) The initial data points could be fitted to a straight line. (**d**) The theoretical (not measurable) rate of heat produced by conversion of two different concentrations of PNPP by *E.coli* alkaline phosphatase. For each concentration of PNPP the graph shows the data points obtained using the Michaelis-Menten equation and the reported K_m_ = 7.8 μM and k_cat_ = 62 (s^−1^). For simulation concentration of *E.coli* alkaline phosphatase was assumed 5 nM. The black line shows a fit to a straight line. The recorded powers of enzymatic reactions (**a**–**c**) were corrected for that of dilution of substrate in the absence of enzyme.

We tested if the data recorded by ITC for the early stages of a reaction, which have been ignored in many previous methods of ITC data analysis for enzyme kinetics^30^,^31^,^32^,^33^, are affected by the rate of enzyme-catalyzed reactions. To this goal we recorded the ITC power signal for the exothermic conversion of different amounts of PNPP by alkaline phosphatase (Fig. 3a), and the power for conversion of PNPP by alkaline phosphatase was corrected for the power recorded as a result of dilution of PNPP in buffer, i.e. in the absence of enzyme, (Methods). For all concentrations of PNPP the lag phase, as explained above, was observed (Fig. 3a). The first data point following the lag phase was considered as the first measurement point, i.e. time = 2 seconds, and the timescale was adjusted accordingly (Fig. 3b). We noticed that for the data points immediately after the lag phase as the rate of enzymatic reaction increased the difference between the recorded powers of two subsequent time points (ΔP_ITC_) increased (Fig. 3b). In other words the larger the initial rate of enzymatic reaction the larger the ΔP_ITC_. This observation shows that the heat transfer and the heat detection rates in the instrument are not the rate limiting step. To explain this observation we wrote the general energy balance equation for the sample cell (Fig. 2c):


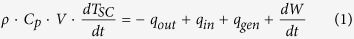


in which 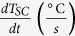 is the temperature change inside the sample cell for a specific sampling time, *C*_*P*_ 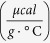, and 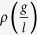 are the heat capacity, and density of the solution inside the sample cell respectively, *V*(*l*) is the volume of the sample cell, 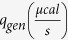 is the rate of heat generated in the sample cell and in the case of an enzyme is equal to *q*_*Enz*_ (Fig. 2c), which includes the rate of heat generated by all the processes associated with the enzymatic turnover such as substrate binding, conformational changes, conversion of substrate to product, product release, and etc., *q*_*out*_ and *q*_*in*_ 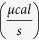 are the rate of heat transfer from the solution inside the sample cell to the outer surface of the sample cell and vice versa respectively (Fig. 2c), and 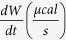 is the change of the work performed by the feedback heater, which is equal to the difference between the recorded power of two subsequent data points (ΔP_ITC_) (Fig. 3b). Equation 1 implies that for the early data points after the lag phase the change in the recorded power (ΔP_ITC_) is a measure of the temperature change in the sample cell, i.e. the more the temperature inside the sample cell changes, the more the change in the recorded power will be. The temperature change inside the sample cell is affected by heat accumulation, which is related to the rate of heat produced (consumed) by the enzymatic reaction (q_Enz_). Thus, the ΔP_ITC_ for the early data points after the lag phase is correlated to the initial rate of the enzymatic reaction.

We noticed that for the lowest substrate concentration the early data points for the first 10–12 seconds after the lag phase, i.e. 5–6 data points (t = 2, 4, 6, 8, 10, and 12 seconds) for a sampling time of 2 seconds, could well be fitted to a straight line (R^2^ > 0.99) (Fig. 3c). As the amount of substrate increased a linear equation could be fitted to more data points (Fig. 3c). This is consistent with the theoretical prediction. We used the reported K_m_ and k_cat_ values for *E.coli* alkaline phosphatase^39^ and applied the Michaelis-Menten equation to predict the theoretical (not measurable) heat flow rate generated by the enzyme for conversion of two different concentrations of PNPP (Fig. 3d). For the lower concentration of PNPP at least five data points could be fitted to a straight line (R^2^ > 0.99) (Fig. 3d), and as the concentration of substrate increased the number of data points that could be fitted to a straight line increased (Fig. 3d). Thus, although ΔP_ITC_ for the early data points after the lag phase and the actual rate of heat flow caused by the enzyme are not equal, they must be linearly related. In other words:


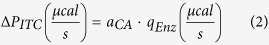


in which *a*_*CA*_ is named the calibration constant. Equation 2 states that the calibration constant defines the difference between the initial response of ITC instrument and the actual heat generated by the enzymatic reaction.

### The calibration constant can be obtained using a calibration reaction

**Figure 4:**
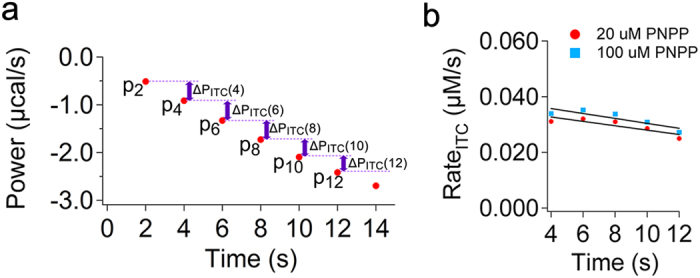
Using a calibration reaction to obtain the calibration constant. (**a**) Early data points after the lag phase recorded for conversion of 100 μM PNPP by alkaline phosphatase (5 nM). The lag phase is removed and data are shifted in time. The first measurement point after the lag phase is P_2_, or power recorded at t = 2 s. The sampling time is 2 seconds. The ΔP_ITC_(t) for each two subsequent data points is shown in the figure. The power recorded by ITC for conversion of PNPP by alkaline phosphatase is corrected for the power recorded due to heat of dilution of PNPP in the absence of enzyme. (**b**) The Rate_ITC_(t) data points obtained from ΔP_ITC_(t) for conversion of two different concentrations of PNPP, i.e. 20 and 100 μM, are plotted as a function of time. Data were fitted to a line (black). The initial Rate_ITC_ is obtained from the y-interception of the line.

We found that for the data points immediately after the lag phase, the ΔP_ITC_ (Fig. 4a) and the initial rate of heat generated by enzyme (q_Enz_) are linearly related. Thus, if we can calculate the constant that defines this linearity, i.e. a_CA_ in equation 2, we can directly convert the early data recorded by ITC to the rate of enzyme, which can then be used to find the initial rate of reaction. To calculate a_CA_ we wrote:


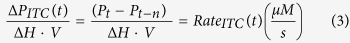


in which *P*_*t*_ is the power recorded at time t, and *P*_*t–n*_ is the power recorded n seconds before t (Figs 3b and 4a), where n is the sampling time, which in our measurements was 2 s, Δ*H* is the enthalpy 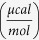 of reaction and accounts for the heat generated by all the processes that occur during the enzymatic turnover including substrate binding, enzyme conformational changes, substrate conversion, product dilution, proton release, and etc., and *V* (liter) is the volume of the sample cell. We also know that:


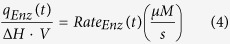


Using equations (2), (3) and (4) for the early data points after the lag phase (Fig. 4a) we may now write:


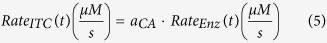


and accordingly we can write:


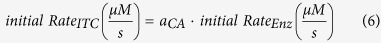


To calculate a_CA_ we used a calibration reaction to obtain the initial Rate_Enz_ by UV-visible spectroscopy and the initial Rate_ITC_ by isothermal titration calorimetry under exactly the same experimental conditions, i.e. the same concentrations of enzyme and substrate, temperature, and buffer. As a calibration reaction we chose the exothermic conversion of 20 or 100 μM PNPP by alkaline phosphatase. We first determined the initial rate of the enzymatic reaction (initial Rate_Enz_) with UV-visible spectroscopy (Methods). To obtain the initial rate of ITC (initial Rate_ITC_) we recorded activity of alkaline phosphates using ITC. For the early data points after the lag phase, which could be fitted to a line (t = 2, 4, 6, 8, 10, and 12 seconds), ΔP_ITC_(t) was obtained (Fig. 4a), e.g. ΔP_ITC_(4) = P_4_ - P_2_. Separately, the ΔH of reaction was found as explained in the methods from a single injection experiment (Supplementary Figure 2). Knowing ΔP_ITC_(t), ΔH, and V, equation 3 was used to calculate the Rate_ITC_ at t = 4, 6, 8, 10, and 12 seconds after the lag phase, the Rate_ITC_(t) data were plotted as a function of time (Fig. 4b), the data were fitted to a line (f(x) = a · x + b) (Fig. 4b), and the initial Rate_ITC_, i.e. rate at t Æ 0, was obtained from the y-interception of the linear equation. Substitution of the values for the *initial Rate*_*ITC*_ and the *initial Rate*_*Enz*_ in equation 6 gave a_CA_ = 0.21 ± 0.02 for both concentrations of PNPP. Based on equation 2 a value of less than 1 for calibration constant is consistent with the fact that the response of an ITC instrument is always lagging behind the actual heat flow rate generated by the enzymatic reaction. In fact if ITC instruments were perfect, meaning that they could record and compensate for the heat generated by the enzymatic reaction instantaneously, and that there was absolutely no heat loss the calibration constant would have a value of 1. Then, we combined equations (3) and (5) to derive an expression for calculating the rate of enzymatic reaction directly from the early data points after the lag phase (Fig. 4a):


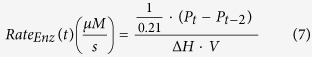


It should be noted that the use of equation 7 requires knowledge of ΔH of a reaction, which can be obtained using a single experiment. This is equivalent to the determination of the molar extinction coefficient of a compound for converting absorbance to concentration using Lambert-Beer law, which is needed to calculate the initial rate (μM/s) of enzymes using colorimetric assays.

### A single calibration constant is valid for various reactions

**Figure 5:**
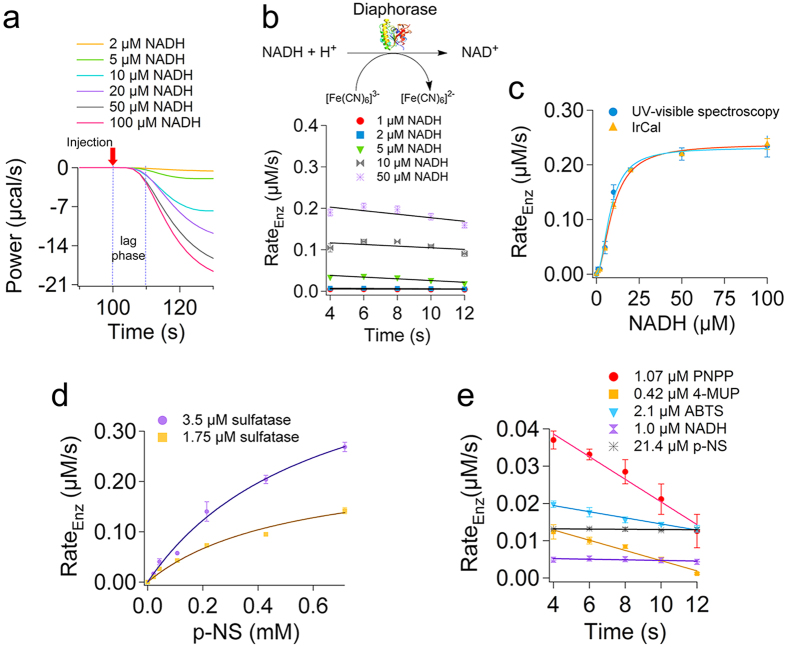
A single calibration constant can be applied to various enzymes. (**a**) The power recorded for conversion of different amounts of NADH by diaphorase. Different amounts of NADH were added before injection of ferricyanide to the sample cell containing 82 nM diaphorase. For each measurement 7 μl of 10 mM ferricyanide, final concentration of 50 μM, as electron accepter was injected in 3 seconds into the sample cell and the power was recorded. The power recorded by ITC for oxidation of each concentration of NADH by diaphorase is corroded for the power recorded due to heat of dilution of ferricyanide. (**b**) For conversion of different amounts of NADH by diaphorase (82 nM) the Rate_Enz_(t) was obtained using equation 7 (text) at t = 4, 6, 8, 10, and 12 seconds after the lag phase. Data were fitted to a line (Black), and the initial rates are obtained from the y-interception of the lines. This method is named IrCal approach. (**c**) The initial rates data obtained using the IrCal approach for different concentrations of NADH are plotted as a function of NADH concentration. These data are compared with the initial rate data obtained using UV-visible spectroscopy. Data were fitted to the Hill equation (the blue and orange lines). (**d**) The initial rate data obtained using IrCal approach for conversion of p-nitrocatechol sulfate (p-NS) in the presence of 1.75 μM or 3.5 μM sulfatase are plotted as a function of p-NS concentration. For each substrate concentration the initial rate increases linearly with the enzyme concentration. Data are fitted to the Michaelis-Menten equation. (**e**) The rate of enzymatic reaction as a function of time is plotted for the lowest concentration of substrates of alkaline phosphatase, diaphorase, sulfatase, and laccase. As the catalytic efficiency (k_cat_/K_m_) of enzyme decreases the slope of a linear fit to the data decreases. Each data point is the average of two measurements ± errors.

We tested if the use of equation 7 and a_CA_ = 0.21 for our ITC instrument, which we obtained using conversion of two specific concentrations of PNPP by alkaline phosphatase as calibration reaction, is valid for other enzymatic reactions. To this goal we measured activity of four different enzymes with various concentrations of their substrates: alkaline phosphatase with PNPP or Methylumbelliferyl phosphate (4-MUP) as two different substrates, sulfatase with p-nitrocatechol sulfate as substrate, diaphorase with NADH as substrate, and laccase with ABTS as substrate. For each enzymatic reaction the power data were recorded for different concentrations of substrate using ITC (Fig. 5a, and Supplementary Figures 3–6), and ΔH of each reaction was obtained as explained for PNPP (Methods). For most concentrations of substrates the heat of dilution was almost negligible (Supplementary Figure 1) and the early ITC data points after correction for heat of dilution were noiseless. Occasionally at high substrate concentrations after correction for heat of dilution, which was large, a noise was observed which died out after 15–20 s (Supplementary Figures 7). Using six data points immediately after the lag phase or after the noise ΔP_ITC_(t) was calculated (Fig. 4a). Subsequently, equation 7 was used to calculate the Rate_Enz_ at t = 4, 6, 8, 10, and 12 seconds after the lag phase or the noise (Fig. 5b and Supplementary Figures 7–11). The Rate_Enz_ vs. time data points were then fitted to a linear equation (f(x) = a · x + b) (Fig. 5b, and Supplementary Figures 7–11), and the y-interception of the line was used as the initial rate, or rate at t Æ 0. The t = 0 is the end of the lag phase. We name this approach, which is to use equation 7 to convert the early data recorded by ITC to the rate of enzyme and to obtain the initial rate from these data, the initial rate calorimetry (IrCal). For each enzyme tested the initial rates obtained using IrCal approach were then plotted as a function of substrate concentrations (Fig. 5c and Supplementary Figures 12–15). To check the accuracy of the IrCal approach we recorded the progress curves for each enzymatic reaction using UV-visible or fluorescence spectroscopy (Methods) (Supplementary Figures 16–20) and the initial rates were obtained as explained in the methods. The initial rate data obtained using spectroscopy were plotted as a function of substrate concentration and were compared to those obtained using IrCal approach (Fig. 5c and Supplementary Figures 12–15). It can be seen that the initial rate data obtained using IrCal approach and UV-visible or fluorescence spectroscopy are within experimental error the same, and when fitted to the enzyme kinetic equations (the Hill equation for diaphorase (Fig. 5c) or the Michaelis-Menten equation for all other enzymes (Supplementary Figures 12–15)) afforded the same enzyme kinetics parameters (Table 1). For sulfatase we also tested whether the initial rates obtained by the IrCal approach change linearly with the enzyme concentration (Fig. 5d). For each concentration of p-nitrocatechol sulfate (p-NS) substrate, a two-fold increase in the concentration of sulfatase led to a two-fold increase in the initial rate of enzymatic reaction (Fig. 5d). Furthermore, from enzyme kinetics we know that for substrate concentrations less than the K_m_, the larger the catalytic efficiency of an enzyme, i.e. k_cat_/K_m_, the faster the substrate will be consumed, and due to substrate depletion the more the rate of enzymatic reaction in the early stages will decrease over time. Thus, for the lowest substrate concentration of each enzymatic reaction that we measured (Table 1), we plotted the Rate_Ezn_(t) data as a function of time (Fig. 5e). It can be observed that for conversion of PNPP by alkaline phosphatase, which has the highest catalytic efficiency (k_cat_/K_m_) (Table 1), the rate decreases more as a function of time and thus, the absolute value of the slope of the linear fit to the Rate_Enz_ versus time is the largest. Consistently, the absolute value of the slope decreases as the catalytic efficiency of enzymes decreases (Fig. 5e). Therefore, we conclude that the IrCal approach is accurate and requires only a single calibration constant (a_CA_). We also compared the enzyme kinetic parameters obtained by the IrCal approach with those we obtained with two previously proposed methods of ITC data analysis^30^ (Table 1), which are not based on the initial rate measurements and are proposed based on the incorrect assumption that the data recorded by ITC are a direct measure of the heat of enzymatic reaction and corrections using the Tian equation^34^,^35^,^36^,^37^,^38^ are not needed. These methods are named baseline shift ITC (BsITC) (Supplementary Figure 21), and (ii) single injection ITC (SiITC)^30^,^40^,^41^,^42^,^43^,^44^,^45^ (Supplementary Figure 22). It can be observed that unlike the K_m_ and k_cat_ obtained with the IrCal approach, those obtained from the BsITC or SiITC methods are not reliable (Table 1), and they differ unpredictably over at least 2 or 3 orders of magnitude from those obtained by UV-vis spectroscopy (Table 1). Therefore, use of previous methods of ITC data analysis with the incorrect assumption that the ITC data are a direct measure of the rate of enzyme will lead to the report of inaccurate and unreliable enzyme kinetics parameters as we observed in previous studies (Supplementary Table 1).

### IrCal approach can be used with natural unlabelled substrates

**Figure 6:**
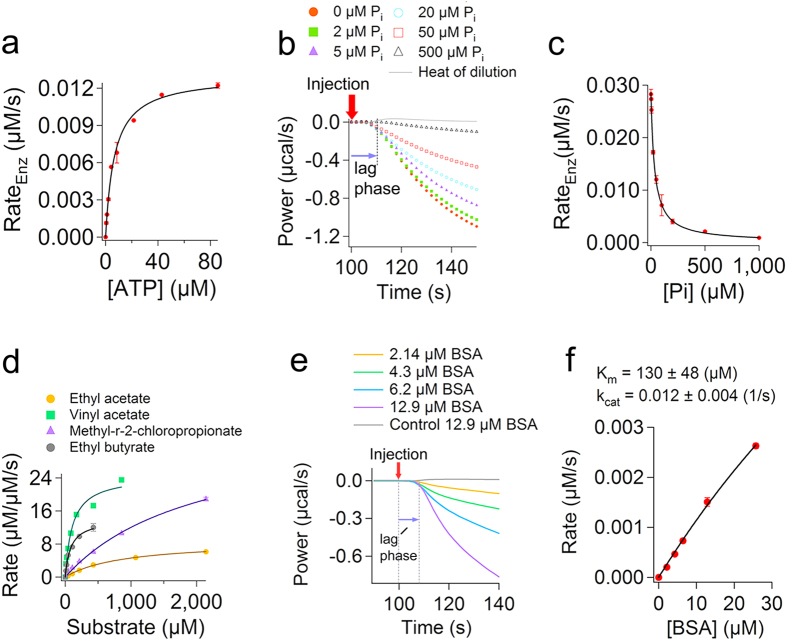
IrCal approach can be used to study enzyme activity with natural substrates. (**a**) The plot of the initial rate data for alkaline phosphatase as a function of different concentrations of ATP as substrate. Concentration of enzyme was 3.7 nM. (**b**) ITC power curves showing the effect of concentration of phosphate on the initial change in the power recorded by ITC for catalysis of 21 μM ATP with 8.5 nM alkaline phosphatase. Data are corrected for the power recorded due to heat of dilution of ATP in buffer (grey line). (**c**) The plot of the initial rate data obtained using the IrCal approach as a function of different amounts of phosphate present before addition of substrate. The solid line shows a fit to v_max_/(1+(I/K_i_)). (**d**) Measurement of the substrate specificity of esterase. Initial rate data (μM substrate converted per μM enzyme per second) are plotted as a function of different substrate concentrations. Concentration of esterase was 0.1 μM for experiments with ethyl acetate, 0.01 μM for those with vinyl acetate, and 0.05 μM for those with methyl-r-2-chloropropionate or ethyl butyrate. (**e**) The recorded power for addition of different amounts of bovine serum albumin (BSA) to pronase e. Each curve represents a single inject of BSA. Concentration of pronase e was 1.4 μM and ΔH of reaction was -158 kCal/mol of BSA added. Data are corrected for the power recorded due to heat of dilution of different amount of BSA in buffer (grey line for dilution of 12.9 μM BSA). (**f**) The plot of the initial rate data obtained using IrCal approach as a function of concentration of BSA. The black line shows a fit to the Michaelis-Menten equation. All measurements were performed at 25 °C. Each data point is average of two measurements ± errors.

We then tested the use of IrCal approach in measuring activity of enzymes using natural unlabelled substrates. Using ITC we recorded conversion of different amounts of ATP by alkaline phosphatase (Supplementary Figure 23), which cannot be measured using UV-visible or fluorescence spectroscopy, and the initial rates were obtained using IrCal approach as explained above (Fig. 6a). From these results the K_m_ and k_cat_ were found to be 6.8 ± 0.6 (μM) and 3.5 ± 0.1 (s^−1^) at pH 7.0. The k_cat_/K_m_ value for ATP, which to our knowledge has not been determined previously, is 0.52 ± 0.03 (1/μM·s). We then, studied the effect of different phosphate concentrations on the rate of conversion of ATP by alkaline phosphatase (Fig. 6b). Phosphate is a known inhibitor of alkaline phosphatase^46^. Initial rate data were obtained using IrCal assay and were plotted as a function of phosphate concentration (Fig. 6c). From the fit of the initial rate data to 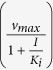, in which I is concentration of inhibitor (μM) and K_i_ (μM) is the inhibition constant, we found that K_i_ = 33.7 ± 1.1 (μM). This value is in good agreement with the values obtained for other alkaline phosphatases using the initial rate measurements of conversion of labelled substrate p-nitrophenyl phosphate^47^,^48^. Then, we tested the use of IrCal assay in measuring the substrate specificity of esterase. Using ITC conversion of four different unlabelled natural substrates were measured (Supplementary Figure 24), and the initial rates at different substrate concentrations were obtained using the IrCal approach. The K_m_ and k_cat_ values for each substrate were obtained from a fit of the initial rate data to the Michaelis-Menten equation (Fig. 6d and Table 2). Different kinetic parameters for each substrate were obtained. Finally, we used the IrCal approach to measure activity of enzymes with large substrates such as proteins or peptides. We recorded activity of pronase e using different concentrations of bovine serum albumin (BSA) as substrate (Fig. 6e), which cannot be measured using other methods, and the initial rate data were obtained using IrCal (Fig. 6f). It should be mentioned that for pronase e the kinetic parameters are apparent because BSA has several possible proteolytic sites and ΔH of reaction (-158 kcal/mol of BSA added) is obtained for complete digestion of BSA by pronase e. The results together explain the applicability of the IrCal approach for fundamental studies of enzymes with natural unlabelled substrates.

## Discussion

We have developed and validated a new approach (initial rate calorimetry, or: IrCal) for reliable label-free determination of the initial rates of enzyme-catalyzed reactions. Briefly, the IrCal approach consists of the following steps: (1) Applying a calibration reaction to obtain a calibration constant for an ITC calorimeter. The calibration constant should be obtained once, and then it can be applied to various other reactions. (2) Using ITC to record the power curves for various reactions. Each curve is obtained for a single addition of substrate similar to the recording of the absorbance using colorimetric assays after a single addition of substrate. After injection of substrate maximum 40 seconds of data recording is needed. The recorded power by ITC for conversion of substrate by enzyme is corrected for the recorded power for dilution of substrate in the absence of enzyme, which in many cases is negligible. (3) After correction of the power curves, the data points immediately after the lag phase, which can be approximated using a line, are used to obtain ΔP_ITC_(t). Subsequently, using the calibration constant of the instrument, and knowing the ΔH of reaction and the volume of the sample cell (V), the values of ΔP_ITC_(t) are converted to Rate_Enz_(t). (4) The Rate_Enz_(t) data are plotted as a function of time, the data are fitted to a line, and the initial rate of enzyme, rate at t Æ 0, is estimated from the y-interception of the line. This approach is based on the first law of thermodynamics (conservation of energy), which is applicable to any ITC instrument. For different instruments only the lag phase and calibration constant, which are properties of the instrument, should be determined. While colorimetric assays require determination of the molar extinction coefficient of the substrates or products to be able to convert the data recorded using UV-visible or fluorescence spectroscopy to the initial rate of enzyme, the IrCal approach requires accurate determination of ΔH of reaction, which can be found by a single experiment. As a result the sensitivity of the IrCal approach can be compared to those of spectroscopic assays. In fact for all the enzymes we tested the same amount of enzyme used in the spectroscopic assays was sufficient to measure the initial rate of enzyme using the IrCal approach (Table 1). The advantage of the IrCal approach is that because heat is a generic property of many reactions unlike spectroscopic assays, which are limited to the substrates that generate a color or fluorescence, it can be applied to various enzymes and natural unlabelled substrates without the need for developing new assays. However, regarding the enzymatic reactions that can be measured limitations exist. In some cases the reaction is entropically driven and thus ITC cannot be used to follow the reaction. Furthermore, the heat of dilution of substrate might be significantly larger than the rate of heat generated by an enzymatic reaction; this will disturb the initial data recorded hugely and as a result these data might not be usable. The IrCal approach however, is not limited to the maximum power that can be measured using ITC. This is because the initial rate measurements using the IrCal approach require data recording for few seconds before the recorded power saturates, which is similar to the initial rate measurements using spectroscopic assays by recording the absorbance for few seconds before it saturates. In regard to the rate of enzymatic reactions there is no upper limit, because the IrCal approach is based on the initial rate of heat accumulation. The higher the rate of a reaction the more heat will accumulate and will be measured. The lower limit however, is when the rate of heat generated by a reaction is as slow as the instrument response time (between 20–100 seconds). For the IrCal method it is preferable that the ITC instrument is set to no-feedback mode to decrease the effect of heat of dilution (Supplementary Figure 25), and to collect more data points during the initial change in the power. Furthermore, the sampling time might be decreased to obtain more data points. In the IrCal approach data after the initial lag phase are not affected by mixing. This conclusion is confirmed by the observation that as more substrate was injected to the cell the initial rate data obtained using IrCal approach for various enzymes were within experimental error the same as those obtained using UV-visible spectroscopy. Thus, full mixing is achieved during the lag phase. Moreover, it should be noted that the initial rates (μM/s) determined using the IrCal method are not affected by the heat of processes such as substrate binding, product dilution, proton release, or enzyme conformational changes. This is because the ΔP_ITC_(t), which is caused by the heat generated due to all the processes associated with the enzymatic turnover, is converted to the rate of enzyme (Rate_Enz_ (t)) using the ΔH of reaction (equation 7), which also accounts for all the processes related to the enzymatic turnover.

The IrCal approach that we have developed does not suffer from some of the limitations of the previously proposed methods for analysis of ITC data^30^,^31^,^32^,^33^. Application of these methods requires use of kinetic models to simulate the full power curves measured by the ITC instrument and thus, data recording for several minutes or sometimes hours is essential to obtain the complete power curves for simulation to be done. This not only is unsuitable for high throughput applications but also causes serious limitations. The substrates or products might not be stable in the long time-scale of measurements. They might decompose, which will generate heat. The products might be further catalyzed by enzyme, generating extra heat. Therefore, simulation of the curves will require knowledge of other reactions that occur and produce heat. For example, in the case of ABTS oxidation we observed a baseline shift (Supplementary Figure 6). While UV-visible spectroscopy showed that oxidation of 2 μM ABTS was complete within 30 seconds (Supplementary Figure 18), the recorded curve by ITC shifted from the original baseline and remained constant even after more than 600 seconds (Supplementary Figure 6). This was possibly due to the heat production because of the instability^49^ and reactivity of the ABTS^.+^ cationic radical toward other components in the reaction mixture, for example amino acids^50^. Therefore, use of previous methods^30^,^31^,^32^,^33^ requires knowledge of the kinetics of reactions of ABTS^.+^ cationic radical. In the case of pronase e activity measurements using BSA as substrate the cleavage of the initial proteolytic sites in BSA will results in the availability of other sites. Each site is cleaved with different rates and thus, simulation of ITC curves using previous methods to obtain kinetics of a proteinase using natural protein substrates requires knowledge of the kinetics of cleavage of each site separately. In contrast the IrCal method is only dependent on the kinetics of the initial reactions.

In conclusion, because heat is a label-free property of many reactions, the IrCal approach has the potential to be used for label-free kinetic studies of different enzymes for which the heat of dilution of substrate is not much greated than the heat generated by the enzymatic reaction. Because the initial rate measurements using the IrCal approach require a few seconds of data recording, this method holds promise for applying the recently developed and fully automated ITC instruments, which are fully automated and have high throughput capability (circa 6–10 samples per hour)^51^, in different areas in which high throughput screening of enzyme activity is required: e.g. discovery of new inhibitors of enzymes like kinases, phosphatase, proteases, or lipid metabolic enzymes, which are important drug targets^13^,^14^,^15^,^52^, as possible drug candidates, or screening of libraries of enzyme mutants for identifying the mutants with improved activity. Furthermore, because heat is produced or consumed as a result of many biological processes such as protein folding or transport of small molecules across membranes, initial rate measurements using the IrCal approach might have the potential to be applied to study the kinetics of these processes.

## Methods

### Enzymes and reagents

All chemicals were reagent grade and were purchased from Sigma Aldrich. Sulfatase type V from *Patella vulgate*, alkaline phosphatase from bovine intestinal mucosa, pig liver esterase, and diaphorase from *Clostridium kluyveri* were also purchased from Sigma Aldrich. Laccase from *Trametes versicolor* was purchased from Fluka. Pronase e from *aus Strptomyces griseus* was obtained from Merc.

### Measuring progress curves using UV-visible or fluorescence spectroscopy

Progress curves were measured using a fiber-optics spectrometer (Avantes) or a Cary spectrofluorometer. For each concentration of substrates 2–10 μl of substrate was added to a glass cuvette with a path length of 1 cm that contained an enzyme solution (final volume 1 ml). Continuous mixing was achieved with a small magnet in the cuvette. p-nitrophenyl phosphate (PNPP) was used as substrate of alkaline phosphatase and the progress curves were recorded at 420 nm for formation of p-nitrophenyl product (Supplementary Figure 16). At 420 nm the molar extinction coefficient of p-nitrophenyl was 12.5 (mM^−1^cm^−1^) (Supplementary Figure 16) (30 °C and pH 8.0). p-nitrocatechol sulphate (p-NS) was used as the substrate of sulfatase and the progress curves were recorded at 515 nm for formation of p-nitrocatechol product. The molar extinction coefficient of p-nitrocatechol at 515 was 0.33 (mM^−1^cm^−1^) (at 45 °C, pH 5.0) (Supplementary Figure 17). For laccase 2,2’-azino-bis(3-ethylbenzothiazoline-6-sulphonic acid) (ABTS) was used as substrate and progress curves were recorded at 600 nm for formation of ABTS^.+^. The molar extinction coefficient of ABTS^.+^ (34 °C and pH 5.8) at 600 nm was 37.2 (mM^−1^cm^−1^) (Supplementary Figure 18). For diaphorase activity measurements NADH was used as substrate and ferricyanide was used as electron acceptor. The progress curves were recorded at 420 nm for reduction of ferricyanide to ferrocyanide and disappearance of the color. The molar extinction coefficient of ferricyanide (25 °C and pH 7.0) at 420 nm was 1.07 (mM^−1^cm^−1^) (Supplementary Figure 19). The progress curves for conversion of different amounts of 4-Methylumbelliferyl phosphate (4-MUP) by alkaline phosphate were recorded using fluorescence spectroscopy (Supplementary Figure 20). Excitation wavelength was 360 nm and emission wavelength was 440 nm. Measurements were performed using a Cary spectrofluorometer at room temperature (ca. 22 °C). The molar extinction coefficient of the 4-Methylumbelliferyl product at 440 nm was 163 μM^−1^cm^−1^, and buffer was 100 mM Mops, 20 mM NaCl pH 7.0.

### Calculating the initial rate of enzymes from the progress curves measured by UV-visible or fluorescence spectroscopy

To determine the initial rate of enzymatic reactions data points from 5–6 seconds after the injection of substrate, when full mixing is achieved, were used. For all enzymes except sulfatase, data points for 10–30 seconds after full mixing were used to calculate the initial rate. For sulfatase and p-NS as substrate data points for 300 seconds after full mixing were used to calculate the initial rate. This was because the molar extinction coefficient of p-NS at pH 5.0 and sulfatase activity were very low. For calculation of the initial rates for oxidation of NADH it should be taken into consideration that NADH donates two electrons and ferricyanide accepts one electron. The slope of a line fitted to the initial data points was used to calculate the initial rate of enzymes (μM/s) using the molar extinction coefficients we obtained for each compound. For each enzyme studied the initial rate data were plotted as a function of substrate concentration. For all enzymes tested except diaphorase, the initial rate data were fitted to the Michaelis-Menten equation, for diaphorase the initial rate data were fitted to the Hill equation. The fitting was done using Igor-pro 5.0.5 software.

### ITC experiments for initial rate measurements (IrCal)

ITC experiments were performed using a VP-ITC instrument (MicroCal/Malvern). For IrCal method each experiment is a single inject of substrate into the sample cell comparable to a single addition of substrate to enzyme in spectroscopic assays. After each experiment the content of the sample cell was removed, the sample cell was cleaned carefully, and was refilled with new enzyme solution. For all enzymatic reactions except diaphorase and hydrolysis of ATP by alkaline phosphatase in the presence of phosphate as inhibitor, the sample cell (1.41 mL) was loaded with an enzyme in a buffer and the syringe was filled with a substrate in exactly the same buffer as that of the enzyme. The volume of each injection into the sample cell was 3, 6, 10 μL, or 12 μL and the injection duration was 2, 3, 4, or 5 seconds, respectively. For diaphorase activity measurements, the sample cell was loaded with different amounts of NADH in the presence of diaphorase, and the syringe was loaded with 10 mM ferricyanide as electron acceptor, and for each NADH concentration 7 μL of ferricyanide solution was injected in 3 seconds, at a final concentration of 50 μM. For hydrolysis of ATP by alkaline phosphatase in the presence of different amounts of phosphate, the syringe was loaded with 10 mM ATP and the sample cell was filled with 8.5 nM alkaline phosphate in the presence of different amounts of phosphate. For each concentration of phosphate, 3 μL of ATP was injected in 2 seconds. For all experiments, after filling the syringe with a substrate, a dummy injection was performed to get rid of the head space in the syringe caused by the initial filling. The ITC instrument was put in no-feedback mode, the stirring speed was 502 rpm to obtain rapid mixing, and the initial delay before injection of substrate was 100 seconds. The no-feedback mode was chosen because in this mode the peak due to dilution is minimized (Supplementary Figure 25) and more data is recorded during the initial change in power. For each measurement in the presence of enzyme a control experiment in the absence of enzyme was performed to obtain the recorded power caused by heat of dilution of the substrate, which was loaded in the syringe. The final ITC power curve for conversion of a substrate by an enzyme was obtained from subtraction of the power recorded for heat of dilution of substrate (in the absence of enzyme) from the power recorded for the conversion of substrate in the presence of enzyme. Experimental conditions for each enzymatic reaction were as follow: sulfatase, temperature 45 °C, buffer 200 mM sodium acetate, 20 mM NaCl pH 5.0, and concentration of enzyme 1.75 μM, unless otherwise stated; alkaline phosphatase and p-nitrophenyl phosphate (PNPP) as substrate, temperature 30 °C, buffer 100 mM Tris, 10 mM NaCl pH 8.0, and concentration of enzyme 5 nM; alkaline phosphatase and 4-MUP as substrate, temperature 25 °C, buffer 100 mM Mops, 20 mM NaCl pH 7.0, and concentration of enzyme 7.5 nM; diaphorase and NADH as substrate temperature 25 °C, buffer 100 mM Mops, 10 mM NaCl, pH 7.0, and concentration of diaphorase 82 nM; laccase and ABTS as substrate, temperature 34 °C, buffer 100mM phosphate, 10 mM NaCl pH 5.8, and concentration of enzyme 30 nM; alkaline phosphatase and ATP as substrate, temperature 25 °C, buffer 100 mM Mops 100 mM NaCl pH 7.0, and concentration of enzyme 3.7 nM or 8.5 nM; esterase, temperature 25 °C, buffer 100 mM Mops 100 mM NaCl, pH 7.0, and concentration of enzyme 0.1, 0.05, or 0.01 μM; and pronase e, temperature 25 °C, buffer 100 mM Mops 100m M NaCl, pH 7.0, and concentration of enzyme 1.4 μM. Origin 7.0 software adopted by the manufacturer for analysis of ITC data was used for correction of data for heat of dilution and for baseline. After obtaining ΔP_ITC_(t) from the raw data using Excel, data analysis and curve fittings were done using Igor-pro 5.0.5 software.

### Determining the lag phase for IrITC measurements

After injection of substrate under steady-state conditions, the initial rate of heat generated by an enzyme remains constant. This is because the initial rate of conversion of substrate to product is constant. As a result the initial change in the power that reflects the heat generated by enzymatic reaction should be linear in time. However, this linearity is observed after a few seconds due to instrumental related delays and due to a finite mixing time. To accurately determine the lag phase, the initial data points for analysis should fit to a straight line and any data before these data points should be removed. The graphical procedure to determine this lag phase is shown in Supplementary Figure 26.

### Measuring enthalpy of reaction

To determine the initial rate of reactions using the IrCal approach the precise ΔH of reactions should be known. The ΔH (kCal/mol) of each reaction was calculated from the area under the complete power curved measured by ITC for conversion of a small amount of substrate by an enzyme after correction for heat of substrate dilution. For example for conversion of PNPP by alkaline phosphatase a single injection of 3 μl (in 2 seconds) of PNPP (final concentration of 2.14 μM) into the sample cell containing 0.005 μM alkaline phosphatase was performed and the data were measured until the recorded power by ITC returned to the base line (Supplementary Figure 2). The area under the curve for conversion of PNPP by alkaline phosphatase was obtained. A separate experiment in the absence of alkaline phosphatase was performed to obtain the heat of dilution of PNPP from the area under the curve. The area under the curve for dilution of PNPP was subtracted from the area under the curve for conversion of PNPP by alkaline phosphatase to obtain the ΔH of reaction, which includes all the processes associated with enzymatic turnover such as substrate binding, enzyme conformational changes, product release, proton release, product dilution, and etc.

### ITC experiments according to the previously proposed methods

For the BsITC method^30^ multi-injection of substrate into the enzyme solution was done to calculate the rate using the method described by Gomez *et al.* (2001), and adopted by the manufacturer (MicroCal/Malvern). The syringe was filled with 20 or 50 mM of p-NS and the cell was filled with 1.4 ml of 200 mM sodium acetate, 20 mM NaCl pH 5.0 containing 0.175 μM, 0.35 μM, or 1.4 μM sulfatase. p-NS solution was prepared in the same buffer as that used in the sample cell. Because the k_cat_ calculated from UV-visible spectroscopy is small (0.14 s^−1^), the steady-state condition can be assumed. 10–16 injections of 3 μl p-NS (each injection 2s) were performed. The spacing time between subsequent injections was 80 seconds. Measurements were performed at 45 °C. Analysis of data was performed using the program implemented by the manufacturer based on the method reported by Gomez *et al.* (2001)^30^. A control experiment, injection of substrate into the buffer in the absence of enzyme, was done to obtain the heat of dilution. ITC experiments were performed in no feedback mode or high feedback mode. For experiments using the single injection ITC (SiITC) method, the cell was filled with 1.75 μM sulfatase or 0.005 μM alkaline phosphatase. For experiments with sulfatase the syringe was filled with 100 mM p-NS and 10 μl was injected in 4 seconds. For experiments with alkaline phosphatase the syringe was filled with 250 mM PNPP and 3 μl was injected in 2 seconds. All other conditions were exactly the same as those used for IrCal and UV-visible spectroscopy.

## Additional Information

**How to cite this article**: Ebrahimi, K. H. *et al.* Accurate label-free reaction kinetics determination using initial rate heat measurements. *Sci. Rep.* **5**, 16380; doi: 10.1038/srep16380 (2015).

## Supplementary Material

Supplementary Information

## Figures and Tables

**Figure 1:**
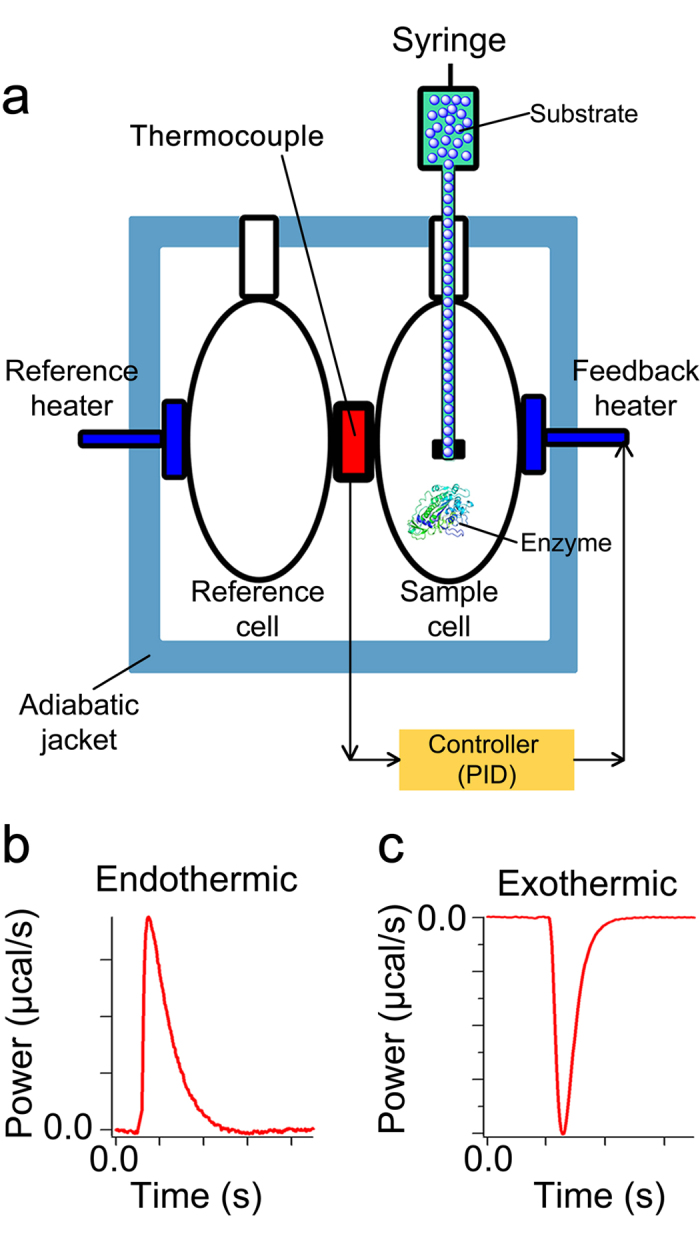
Heat measurements using isothermal titration calorimetry. (**a**) An automated syringe that also acts as stirring tool is used to inject the substrate into the sample cell, which contains the enzyme. The reference cell only contains water. Before injection of substrate into the sample cell, the temperature of the sample cell is the same as that of the reference cell. Moreover, the temperature of the volume of the substrate solution (at least 20 μl) in the part of the syringe that is located in the sample cell, is exactly the same as that of the content of the sample cell. The enzymatic reaction is either exothermic or endothermic, and as a result after injection of substrate the temperature inside the sample cell increases or decreases respectively. The difference between the temperature of the sample cell and the constant temperature of the reference cell is detected by thermocouples. This difference is then translated by a proportional-integral-derivative (PID) controller to the power, or heat flow rate (μcal/s), that should be applied to the feedback heater in order to bring the temperature of the sample cell back to that of the reference cell. The change in the power of the feedback heater relative to the constant power of the reference heater, for short power (P), is recorded as the output signal. (**b**) A typical heat flow rate recorded by ITC for an endothermic and (**c**) an exothermic reaction.

**Table 1:** Comparison of enzyme kinetic parameters obtained with different methods.

Enzyme	Substrate	ΔH (kcal/mol)	Method	T (°C)	μM Enzyme	K_m_(μM)	k_cat_ (s^−1^)	k_cat_/K_m_ (1/μM·s)
Sulfatase	>p-NS	>−12	UV-visible spectroscopy	45	1.75	578 ± 126	0.14 ± 0.01	(2.5 ± 0.4)·10^−4^
			IrCal	45	1.75	507 ± 100	0.14 ± 0.02	(2.3 ± 0.2)·10^−4^
			IrCal	45	3.5	618 ± 136	0.14 ± 0.02	(2.3 ± 0.2)·10^−4^
			BsITC (a)	45	0.175	170 ± 11	0.83 ± 0.02	(4.9 ± 0.2)·10^−3^
			BsITC (b)	45	1.4	92 ± 9	0.14 ± 0.004	(1.5 ± 0.1)·10^−3^
			BsITC	45	0.175	171 ± 12	0.52 ± 0.01	(3.1 ± 0.2)·10^−3^
			BsITC	45	0.35	194 ± 30	0.42 ± 0.02	(2.2 ± 0.2)·10^−3^
			SiITC (c)	45	1.75	561 ± 66	0.46 ± 0.04	(8.2 ± 0.3)·10^−4^
Alkaline phosphatase	PNPP	−8.5	UV-visible spectroscopy	30	0.005	9.31 ± 1.1	46.92 ± 1.1	5.3 ± 0.2
			IrCal	30	0.005	7.78 ± 1.5	47.36 ± 2.0	6.3 ± 0.9
			SiITC (d)	30	0.005	424 ± 5.1	334 ± 3.0	0.79 ± 0.002
	4-MUP	−1.5	Fluorescence spectroscopy	22	0.01	3.20 ± 0.70	7.20 ± 0.40	2.3 ± 0.40
			IrCal	25	0.0075	2.50 ± 0.40	9.50 ± 0.30	2.6 ± 0.3
*Diaphorase	NADH	−33	UV-visible spectroscopy	25	0.08	57.8 ± 8.3	2.8 ± 0.08	0.06 ± 0.01
			IrCal	25	0.08	60.4 ± 8	2.9 ± 0.1	0.05 ± 0.01
Laccase	ABTS	−3.8	UV-visible spectroscopy	34	0.06	42.1 ± 2.2	24.9 ± 0.6	0.59 ± 0.02
			IrCal	34	0.03	46.7 ± 11.3	28.1 ± 2.7	0.62 ± 0.10

Except diaphorase the kinetic parameters for other enzymes were determined by fitting the initial rate data to the Michaelis-Menten equation. The kinetic parameters for diaphorase were obtained by fitting the initial rate data to the Hill equation. For IrCal experiments with laccase the reference power was 1 μcal/s, for all other enzymes the reference power was 12.1 μcal/s. For the baseline shift ITC (BsITC) and single injection ITC (SiITC) methods enzyme kinetic parameters were obtained using the manufacturer’s (MicroCal) protocol and the software designed based on the method proposed by Gomez *et al.* (2001). (a) and (b) were performed in high-feedback mode and all other experiments were done in no-feedback mode. (c) For the SiITC experiment with sulfatase 10 μl of p-NS (100 mM) was injected into the sample cell. (d) For the SiITC method with alkaline phosphatase and PNPP as substrate the concentration of PNPP in the syringe was 250 mM and 3 μl was injected. * Diaphorase showed positive cooperatively with an apparent degree of cooperativity of n = 1.9 ± 0.2 (UV-visible spectroscopy) or n = 1.8 ± 0.2 (IrCal). For diaphorase, ΔH and enzyme activity parameters are per mole of NADH oxidized. Final concentration of ferricyanide used as the electron acceptor in the diaphorase activity measurement was 50 μM.

**Table 2:** Measuring substrate specificity of esterase.

Substrate	Esterase (μM)	ΔH (kcal/mol)	K_m_ (μM)	k_cat_ (s^−1^)
Ethyl acetate	0.10	−5.2	898.0 ± 54.8	8.5 ± 0.23
Vinyl acetate	0.01	−12.2	109.8 ± 21.5	23.8 ± 1.5
Methyl-r-2-chloropropionate	0.05	−5.7	2070 ± 336	36.9 ± 3.6
Entyl butirate	0.05	−4.2	93.9 ± 10.7	14.0 ± 0.6

Activity of esterase was recorded at different substrate concentrations using ITC. For each concentration of substrate the initial rate was determined using IrCal approach and the enzyme kinetic parameters were found by fitting the initial rate data to the Michaelis-Menten equation. All measurements were done at 25 °C.
