# Supplementary material for: Accurate label-free reaction kinetics determination using initial rate heat measurements
Source: Sci Rep. 2015 Nov 17;5:16380. doi: 10.1038/srep16380 (PMC4647221; doi:10.1038/srep16380)
Supplement: Supplementary Information [file srep16380-s1.pdf]

Supplementary Information

Accurate label-free reaction kinetics determination using initial rate heat measurements

Kourosh Honarmand Ebrahimi[1]*, Peter-Leon Hagedoorn[1], Denise Jacobs[2], Wilfred R. Hagen[1]*

[1]Delft University of Technology, Department of Biotechnology, Julianalaan 67, 2628 BC Delft, The Netherlands, [2]DSM Biotechnology Center, Alexander Fleminglaan 1,2613 AX Delft, the Netherlands

Kourosh H. Ebrahimi, Department of Biotechnology, Delft University of Technology, Julianalaan 67, 2628BC Delft, The Netherlands, Phone: 0031152782347, Email: k.h.ebrahimi@tudelft.nl

Wilfred R. Hagen, Department of Biotechnology, Delft University of Technology, Julianalaan 67, 2628BC Delft, The Netherlands, Phone: +31152785051 ; Fax: +31152782355; Email: w.r.hagen@tudelft.nl

Supplementary Tables

Supplementary Table 1. Comparison of literature values for enzyme kinetic parameters obtained by the BsITC or SiITC methods with those obtained by colorimetric methods.

Enzyme	Substrate	Colorimetric Methods		BsITC or SiITC Methods		Ref.
		K_m (uM)	k_{cat} (1/s)	K_m (uM)	k_{cat} (1/s)	
DHFR	DFH	6	3	1.2	6	[1]
Yeast Hexokinase	Glucose	100	450	72	270	[1]
B. cereus penicillinase I	benzylpenicillin	50	2800	120	3600	[1]
HIV protease	VSQNYPIVQ peptide	15	45	5-300	10	[1]
H. pylori urease	Urea	170	2700	790	1400	[1]
F. heparinum heparinise-I	heparine	10.2	92	1.8	0.059	[1]
Rubisco	Ribulose bisphosphate	53	1.76	150	1.95	[1]
Acetolactase synthase	Pyruvate	5.5	5.3	4.8	11	[1]
Pyruvate carboxylase	ATP	58	---	85	---	[1]
Pyruvase carboxylase	Pyruvate	440	---	105	---	[1]
B-glucosidase (Free enzyme)	NPG	5	6.5	4	5.7	[2]
B-glucosidase (Immobilized)	NPG	7	2.8	5	2.3	[2]
B. adusta versatile peroxidase	Lignin peroxidase (LiP)	13	0.18	4	0.37	[3]
B. adusta versatile peroxidase	Manganese peroxidase (MnP)	7	2.92	4	3.88	[3]

Supplementary Figures

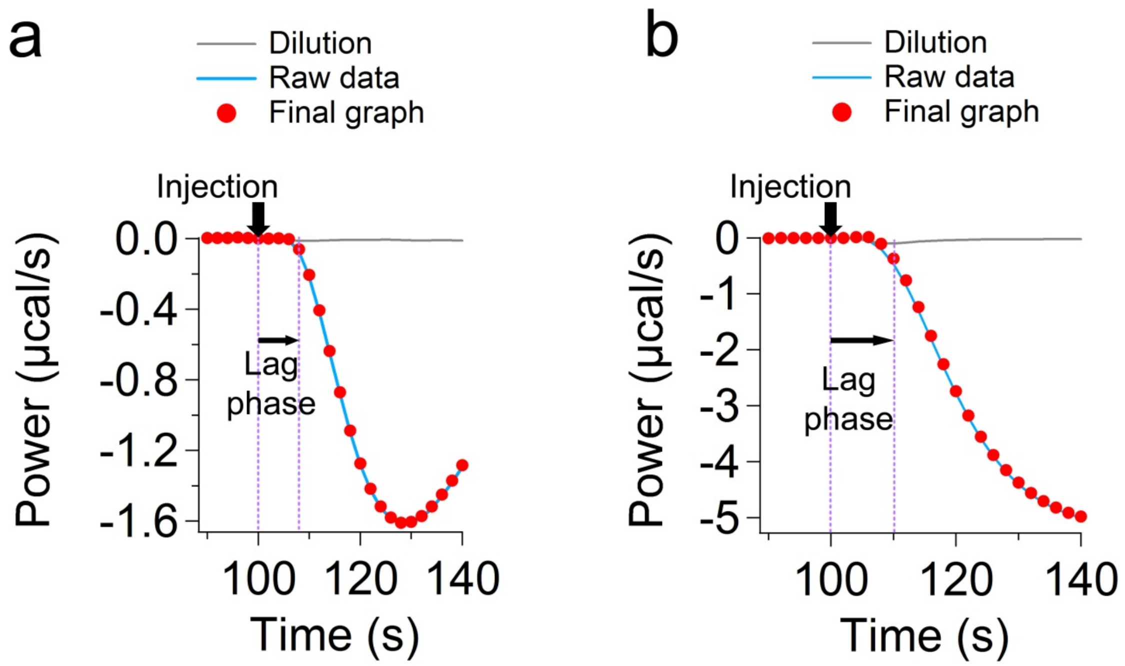

Supplementary Figure 1. The power recorded by ITC for conversion of a substrate by an enzyme should be corrected for the power recorded for the heat of dilution of substrate in the absence of enzyme. The power recorded (a) for a single injection of 3 µl PNPP (2 mM in syringe) in 2 seconds, and (b) for a single injection of 6 µl PNPP (40mM in syringe) in 3 seconds. Both in (a) and (b) the power recorded for conversion of PNPP by enzyme (Raw data, blue line) is subtracted from the power recorded for dilution of PNPP (grey line) to obtain the final graph (red markers). Concentration of alkaline phosphatase was 5 nM. Measurements were performed at 30 °C. The reference power was 12.1 µcal/s.

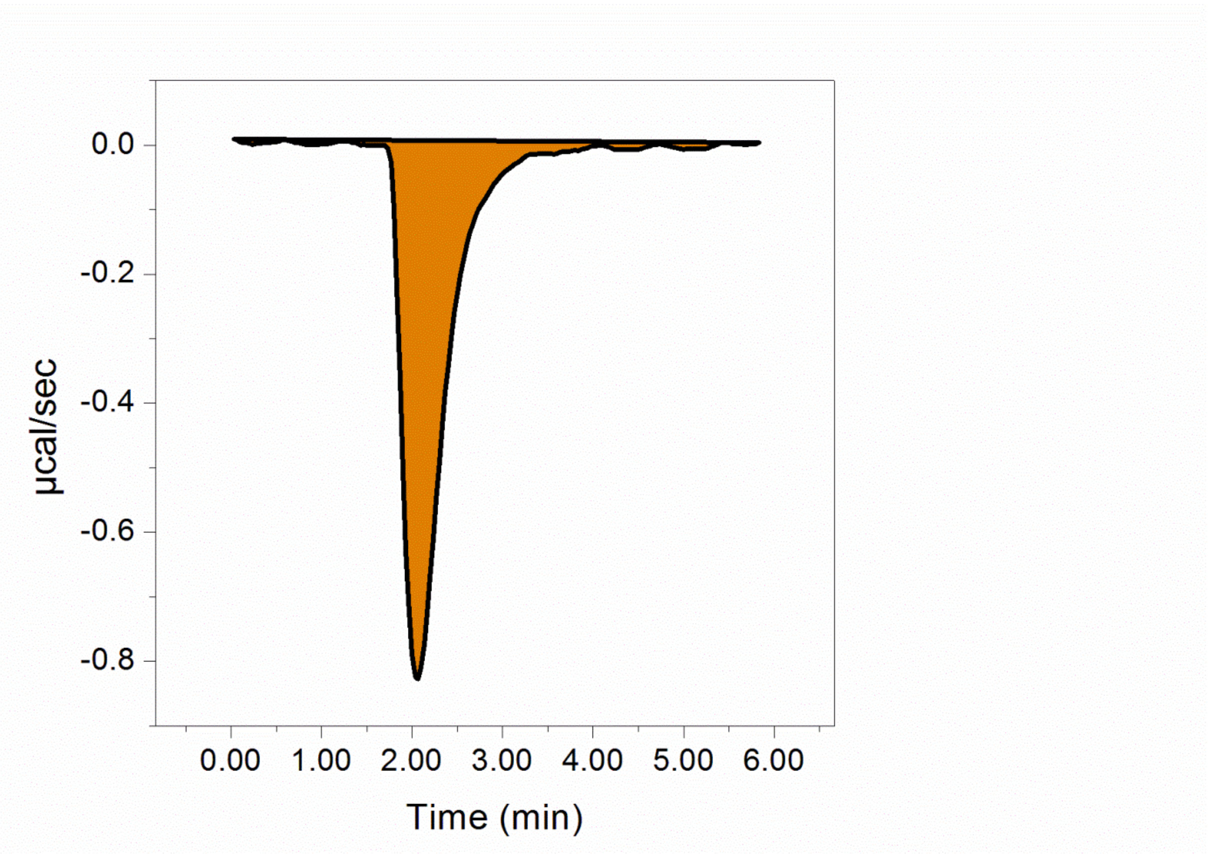

Supplementary Figure 2. ΔH (kcal/mol) of conversion of substrate to product is obtained from the area under the curve. A single injection of 3 µl (in 2 seconds) of PNPP (final concentration of 2.14 µM) into the cell containing 0.005 µM alkaline phosphatase. The data are corrected for heat of dilution. Enthalpy of the reaction was -8.5 kcal per mol of PNPP added. The reference power was 12.1 µcal/s.

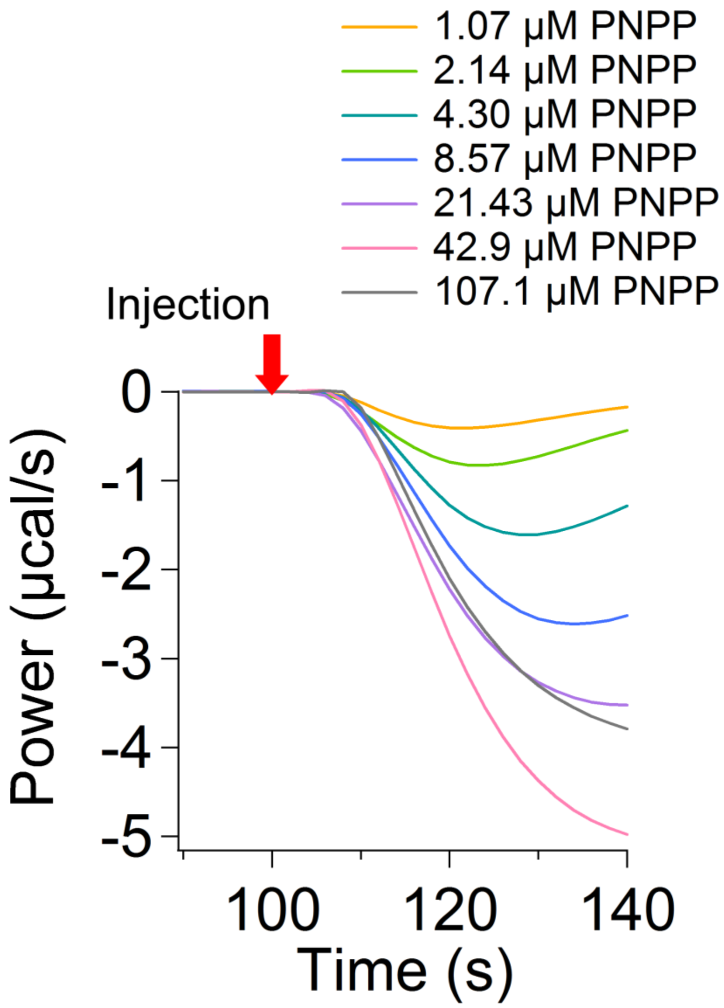

Supplementary Figure 3. The recorded ITC power curves for enzymatic conversion of different amounts of p-nitropheynyl phosphate (PNPP). For final concentrations of 1.07 µM or 2.14 µM PNPP, the syringe was filled with 0.5 mM PNPP and 3 µl (in 2 seconds) or 6 µl (in 3 seconds) of PNPP was injected respectively. For final concentrations of 4.28 µM or 8.57 µM PNPP, the syringe was filled with 2 mM PNPP and 3 µl (in 2 seconds) or 6 µl (in 3 seconds) of PNPP was injected respectively. For final concentrations of 21.4 µM or 42.85 µM PNPP, the syringe was filled with 10 mM PNPP and 3 µl (in 2 seconds) or 6 µl (in 3 seconds) of PNPP was injected respectively. For final concentrations of 107.14 µM or 214.3 µM PNPP, the syringe was filled with 50 mM PNPP and 3 µl (in 2 seconds) or 6 µl (in 3 seconds) of PNPP was injected respectively. Concentration of alkaline phosphatase in the sample cell was 0.005 µM. Measurements were performed at 30 °C. Buffer was 100 mM Tris, NaCl 10 mM, pH 8.0. The reference power was 12.1 µcal/s. The powers recorded by ITC for conversion of each concentration of PNPP by alkaline phosphatase is corroded for the power recorded due to heat of dilution of that concentration of PNPP.

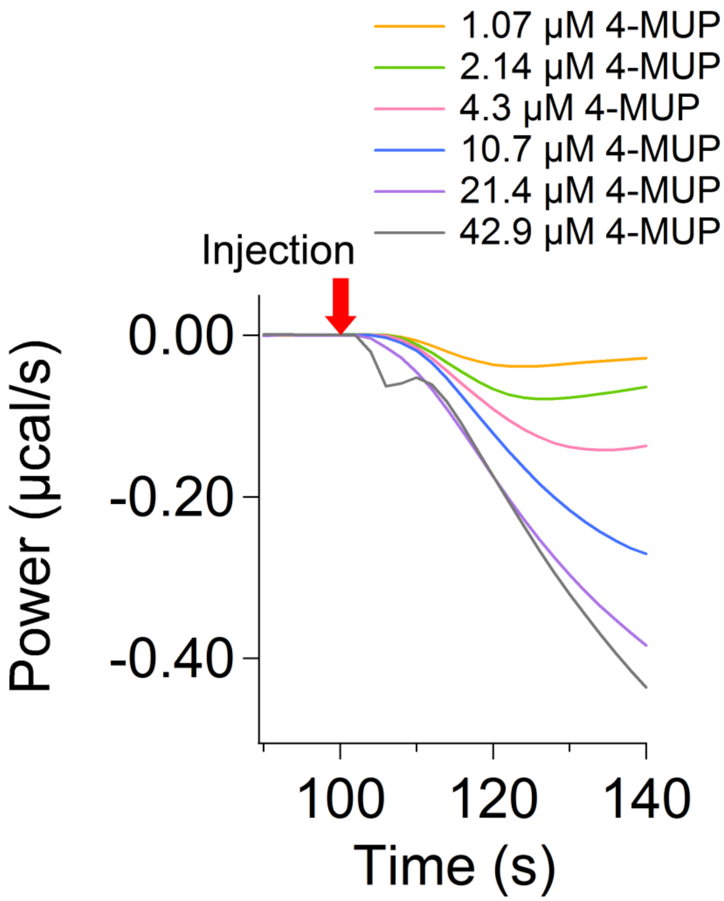

Supplementary Figure 4. The recorded ITC power curves for enzymatic conversion of different amounts of 4-Methylumbelliferyl phosphate (4-MUP) by alkaline phosphatase. For final concentrations of 0.43 µM, 1.04 µM, 2.1 µM, 4.3 µM, 10.7 µM, 21.4 µM, 42.9 µM, or 64.3 µM 4-MUP, the syringe was filled with 0.2 mM, 0.5 mM, 1 mM, 2 mM, 5 mM, 10 mM, 20 mM, or 10 mM of 4-MUP respectively and 3 µl (in 2 seconds) of 4-MUP was injected. Concentration of alkaline phosphatase in the sample cell was 0.0075 µM. Measurements were performed at 25 °C. Buffer was Mops 100 mM, NaCl 20 mM pH 7.0. The reference power was 12.1 µcal/s. The powers recorded by ITC for conversion of each concentration of 4-MUP by alkaline phosphatase is corroded for the power recorded due to heat of dilution of that concentration of 4-MUP.

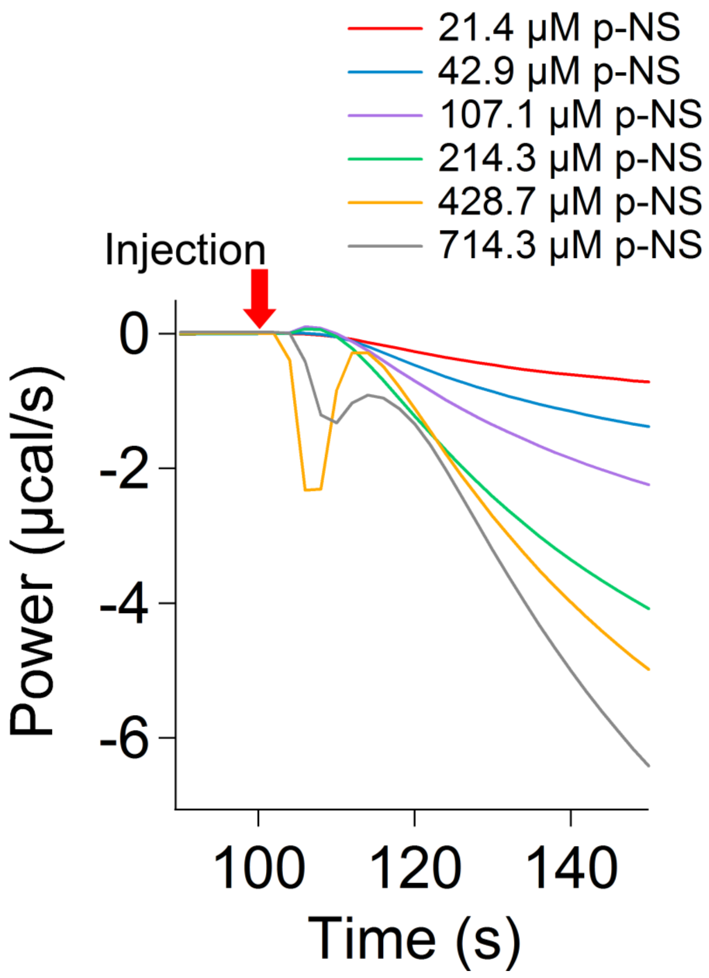

Supplementary Figure 5. Recorded ITC power curves for enzymatic conversion of different amount of p-nitrocatechol sulphate (p-NS) by sulfatase. For final concentrations of 21.4 µM or 42.8 µM p-NS, the syringe was filled with 10 mM p-NS and 3 µl (in 2 seconds) or 6 µl (in 3 seconds) of p-NS was injected respectively. For final concentrations of 107.14 or 214.3 µM p-NS the syringe was filled with 50 mM p-NS and 3 µl (in 2 seconds) or 6 µl (in 3 seconds) was injected. For final concentrations of 428.7 or 714.3 µM p-NS the syringe was filled with 100 mM p-NS and 6 µl (in 3 seconds) or 10 µl (in 5 seconds) was injected. Concentration of enzyme in the sample cell was 1.75 µM. Measurements were performed at 45 °C. The data are corrected for the heat of dilution. For each injection of substrate the sample cell was loaded with fresh enzyme solution. The ITC experiments were stopped before the recorded power reached the baseline because full conversion of substrate took more than 4 hours as observed by UV-visible spectroscopy (Supplementary figure 13). The reference power was 12.1 µcal/s. The powers recorded by ITC for conversion of each concentration of p-NS by sulfatase is corroded for the power recorded due to heat of dilution of that concentration of p-NS.

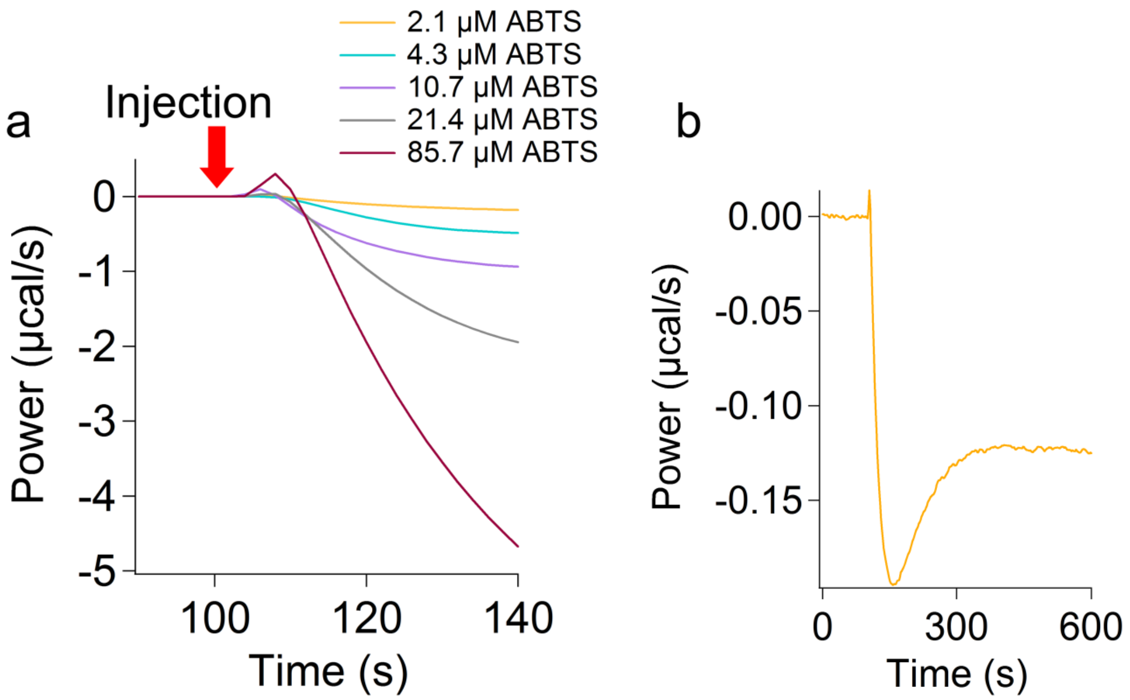

Supplementary Figure 6. The recorded ITC curves for oxidation of ABTS with laccase. (a) Different amounts of ABTS was injected into the sample cell containing 0.03 µM laccase. For 2.1µM and 4.3µM ABTS, 3 and 6 µl of 1mM ABTS solution was injected respectively. For 10.7 and 21.4 µM ABTS, 3 and 6 µl of 5 mM ABTS solution was injected respectively. For 85.7 µM ABTS, 6 µl of 20 mM ABTS solution was injected. Buffer was 100 mM phosphate, 10 mM NaCl pH 5.8. Measurements were performed at 34 °C. The powers recorded by ITC for conversion of each concentration of ABTS by laccase is corroded for the power recorded due to heat of dilution of that concentration of ABTS. (b) The ITC power curve recorded for oxidation of 2.1 µM ABTS by laccase. A drift in the baseline is observed which is possibly because of formation of $ABTS^{\cdot+}$ radical and reaction of this radical with other components in the solution. To obtain enthalpy of reaction, data were corrected for the drift in the baseline.

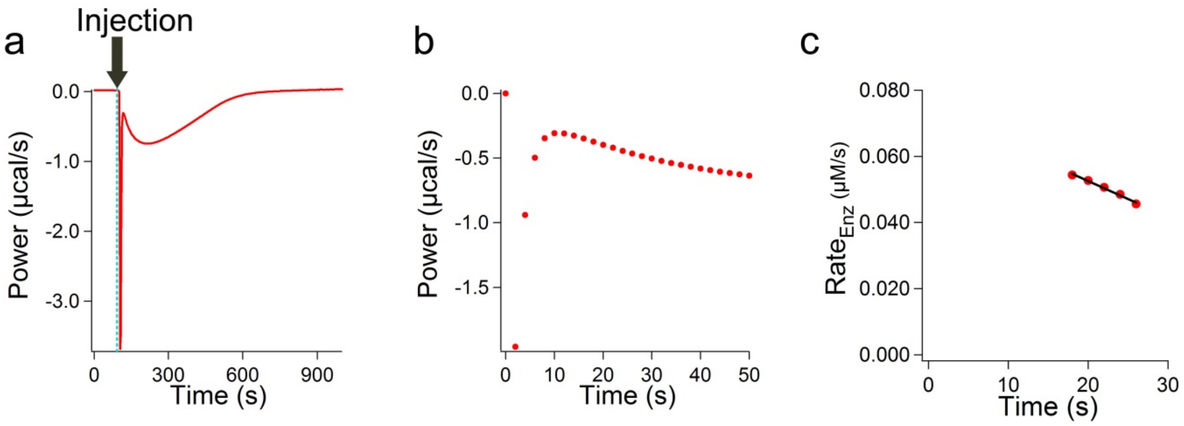

Supplementary Figure 7. Calculation of the initial rate for ITC curves with significant noise in the early stage of the reaction. (a-c) Enzymatic conversion of 4-MUP with alkaline phosphatase. (a-b) The recorded power for conversion of 85.71 µM 4-MUP by alkaline phosphatase (0.0075 µM). The recorded power is shifted for 100 seconds measurements before injection and 8 seconds lag phase after injection of substrate. Significant noise is observed over the next 16 seconds. (c) The calculated rate of enzymatic reaction using equation 7 (Main text) for data points from 18-24 seconds which could be fitted to a straight line $R^2 > 0.99$. The initial rate (rate at t=2 s) was estimated from the fit of a line to the data points.

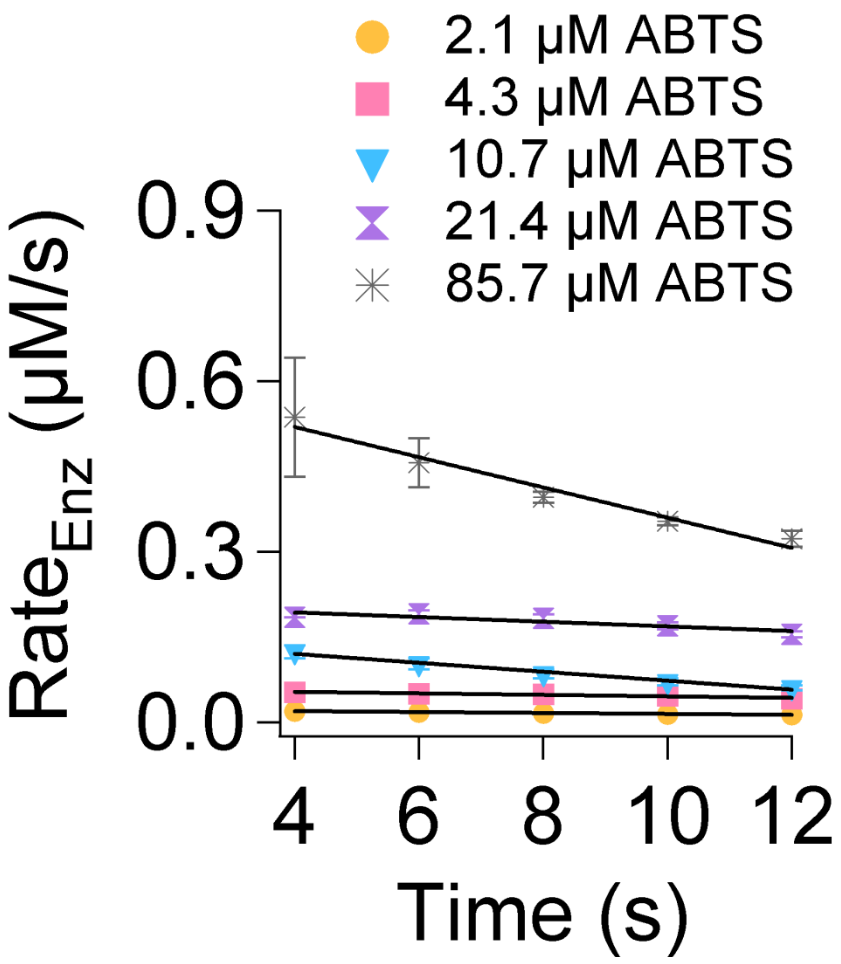

Supplementary Figure 8. The Rate$_{Enz}$ for oxidation of different amounts ABTS by laccase are plotted as a function of time. For each concentration of ABTS Rate$_{Enz}$ was obtained using IrCal approach for five different time points, t = 4, 6, 8, 10, and 12 seconds after the lag phase. The reference power was 1 µcal/s. Measurements with ABTS were done at 34 °C. Concentration of laccase in the cell was 0.03 µM.

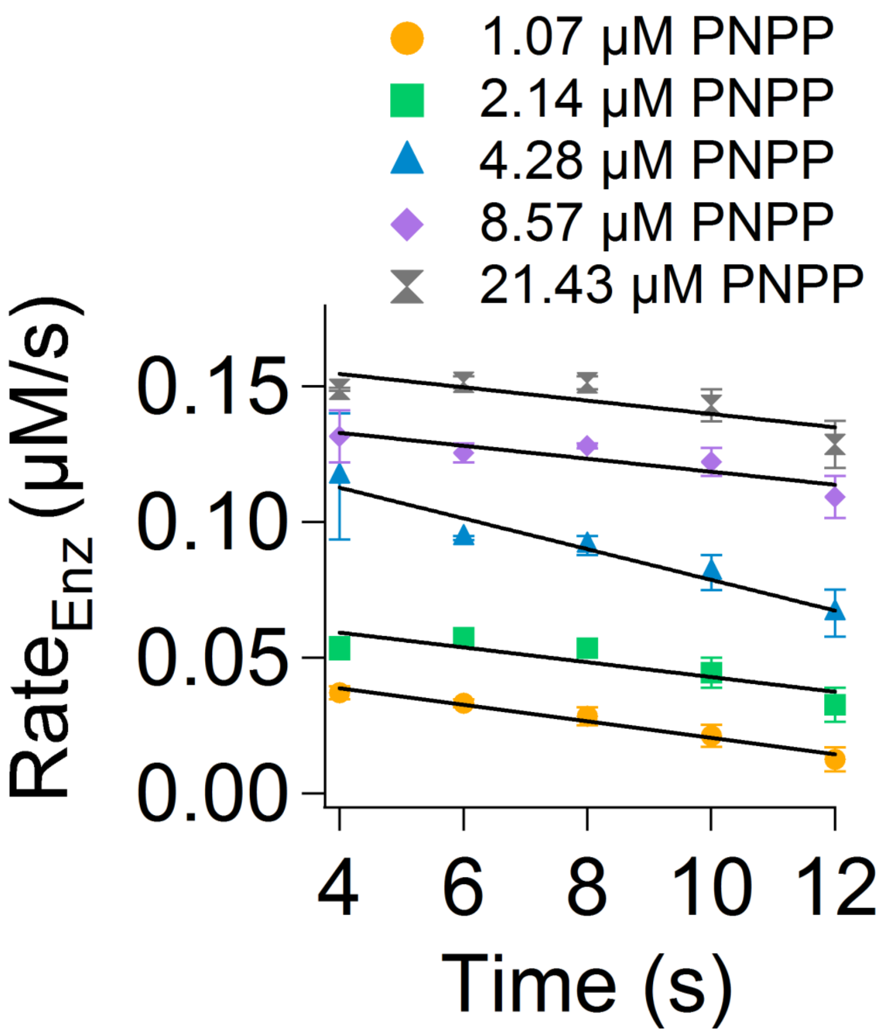

Supplementary Figure 9. The Rate$_{Enz}$ for conversion of different amounts PNPP by alkaline phosphatase are plotted as a function of time. For each concentration of PNPP Rate$_{Enz}$ was obtained using IrCal approach for five different time points, t = 4, 6, 8, 10, and 12 seconds after the lag phase. The reference power was 12 μcal/s. Measurements with PNPP were done at 30 °C. Concentration of alkaline phosphatase in the cell was 5 nM.

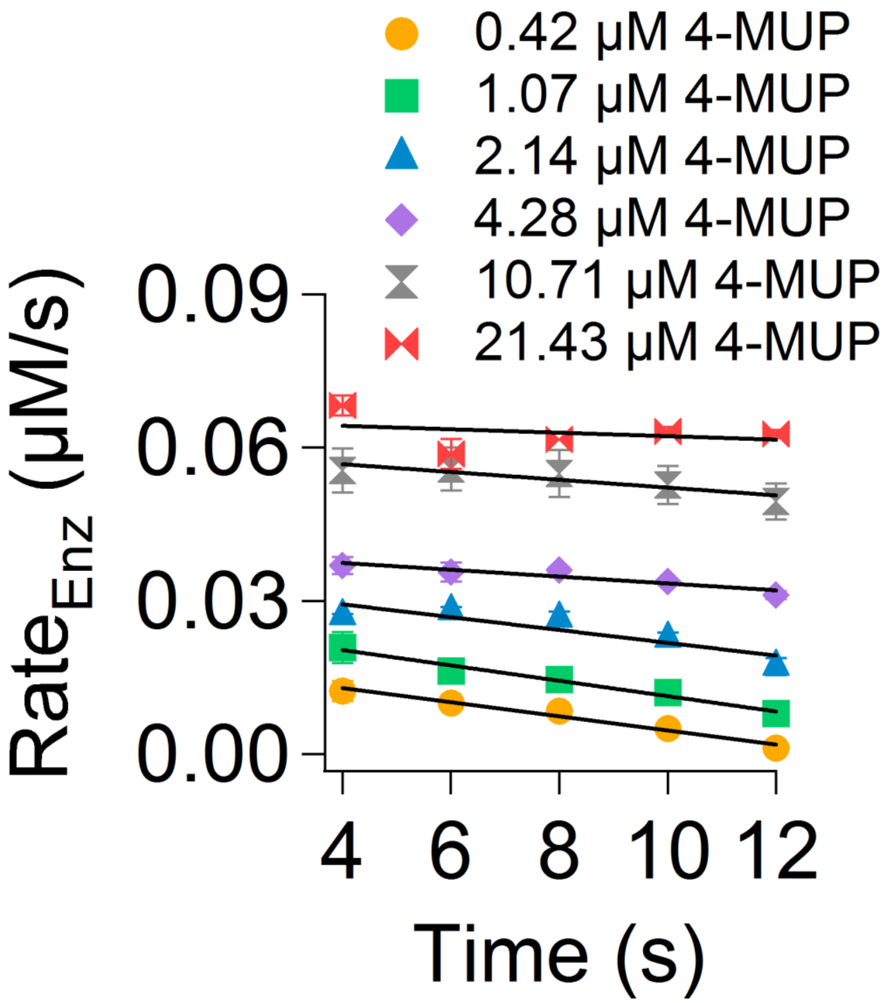

Supplementary Figure 10. The Rate$_{Enz}$ for conversion of different amounts 4-MUP by alkaline phosphatase are plotted as a function of time. For each concentration of 4-MUP Rate$_{Enz}$ was obtained using IrCal approach for five different time points, t = 4, 6, 8, 10, and 12 seconds after the lag phase. The reference power was 12 µcal/s. Measurements with 4-MUP were done at 25 °C. Concentration of alkaline phosphatase in the cell was 7.5 nM.

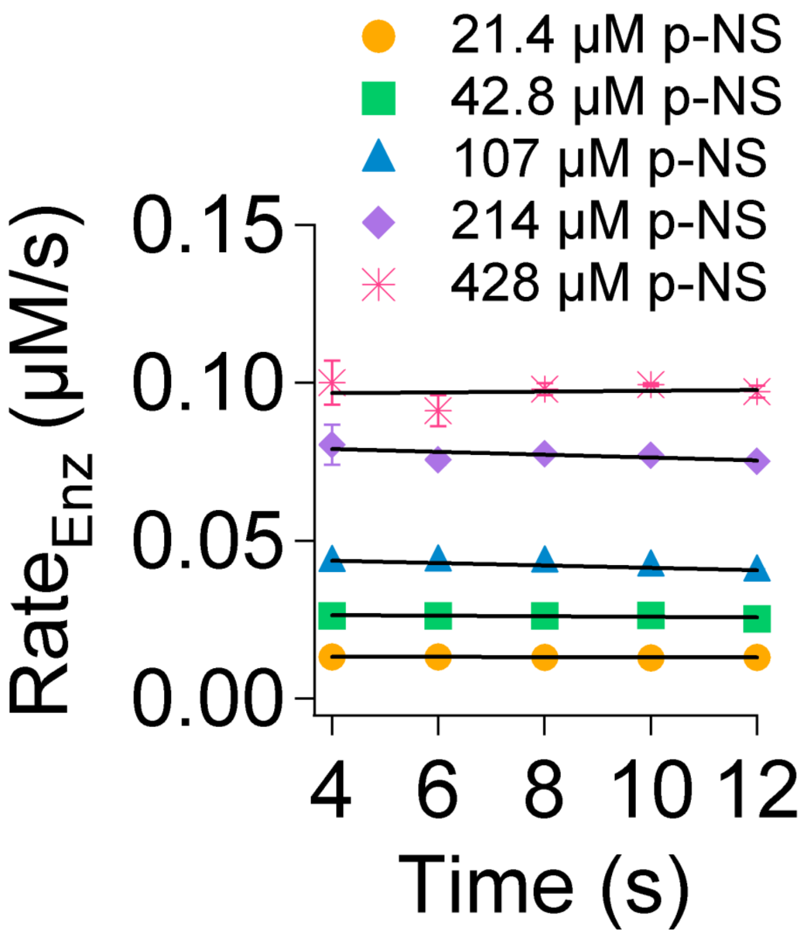

Supplementary Figure 11. The $Rate_{Enz}$ for conversion of different amounts p-NS by sulfatase are plotted as a function of time. For each concentration of p-NS $Rate_{Enz}$ was obtained using IrCal approach for five different time points, t = 4, 6, 8, 10, and 12 seconds after the lag phase. The reference power was 12 µcal/s. Measurements with p-NS were done at 45 °C. Concentration of sulfatase in the cell was 1.75 µM.

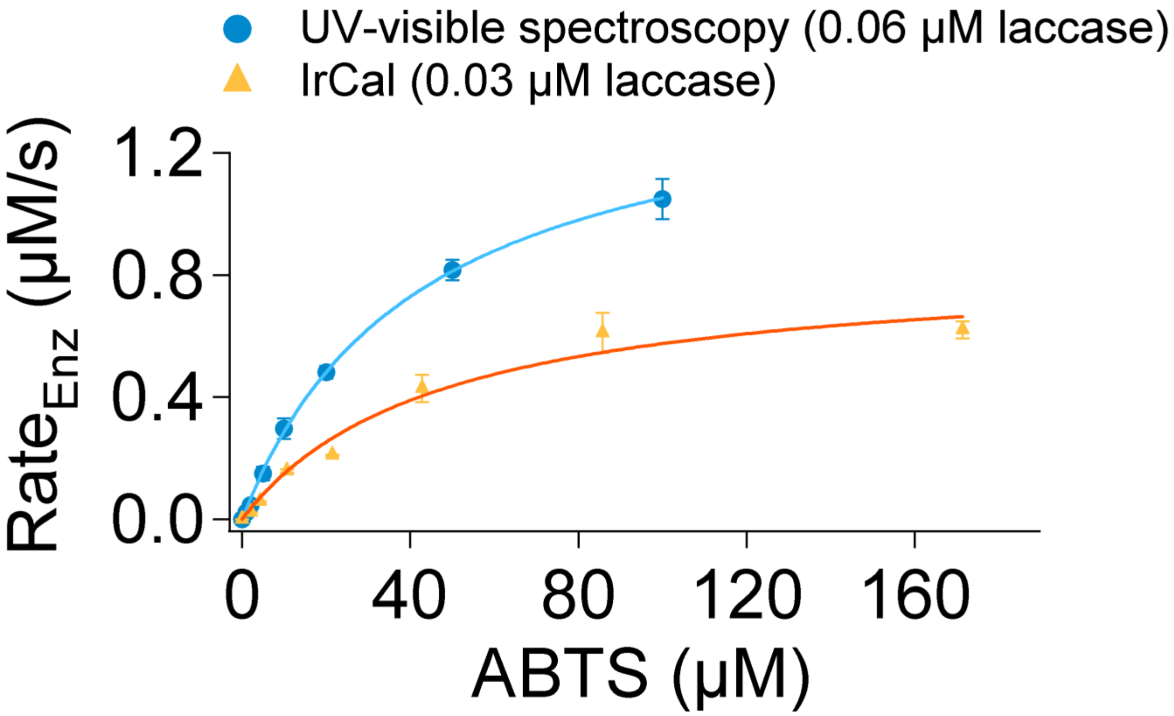

Supplementary Figure 12. The plot of the initial rate as a function of substrate concentration for oxidation of ABTS by laccase. Concentration of laccase for UV-visible spectroscopy measurements was 0.06 µM and for IrCal measurements was 0.03 µM. The fits to the Michaelis-Menten equation give the same K_m and k_{cat} values (See Table 1). Measurements were performed at 34 °C.

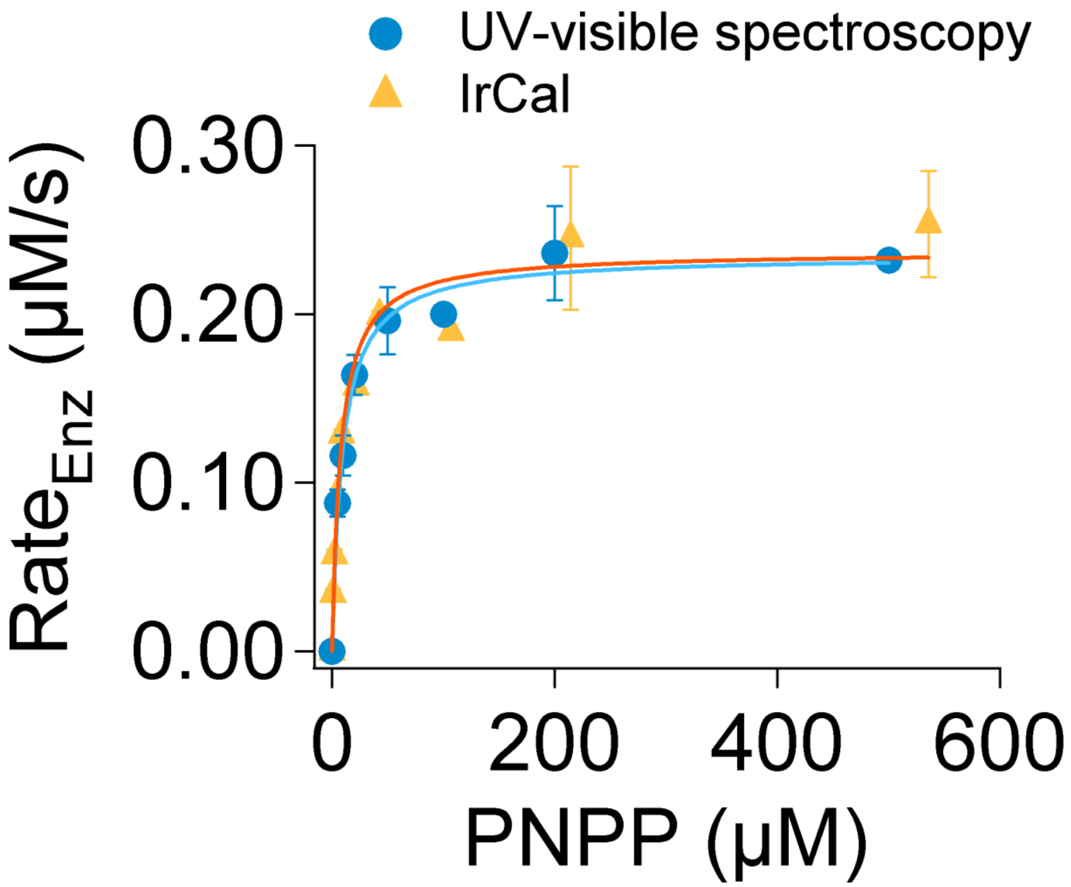

Supplementary Figure 13. The plot of the initial rate as a function of substrate concentration for conversion of PNPP by alkaline phosphatase.

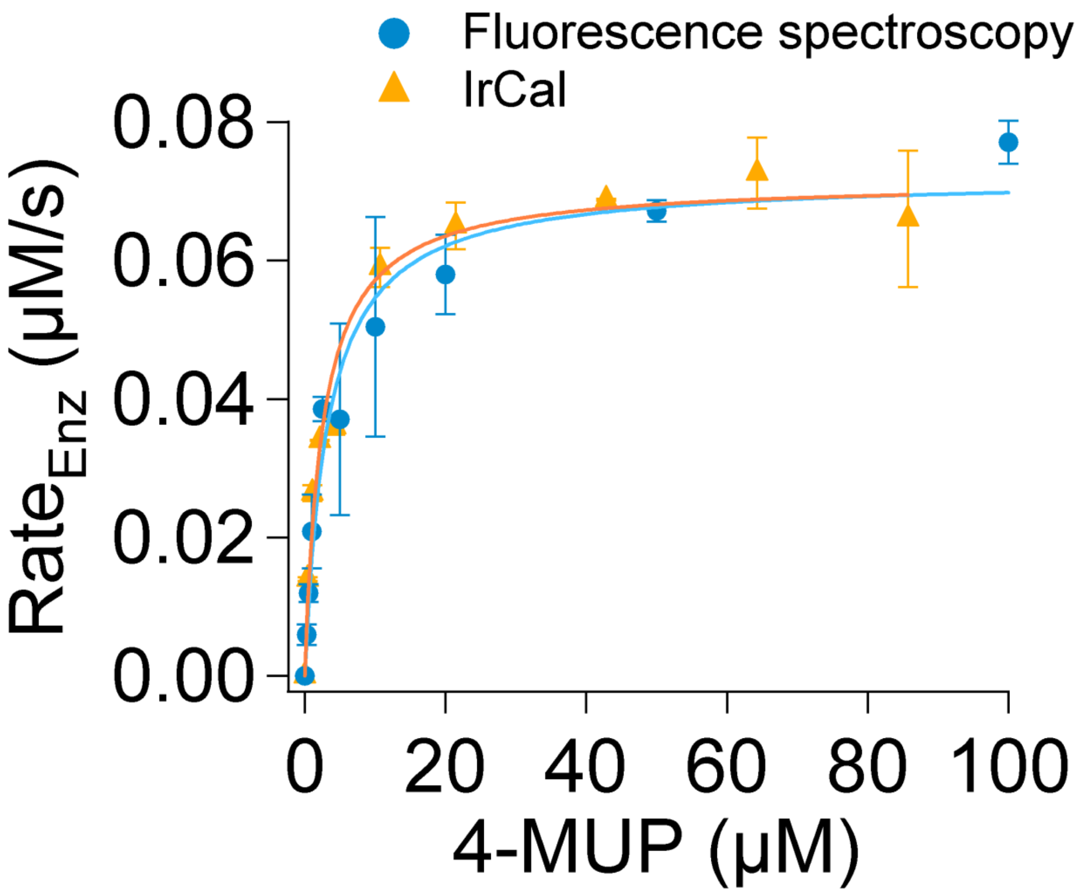

Supplementary Figure 14. The plot of the initial rate as a function of substrate concentration for conversion of 4-MUP by alkaline phosphatase.

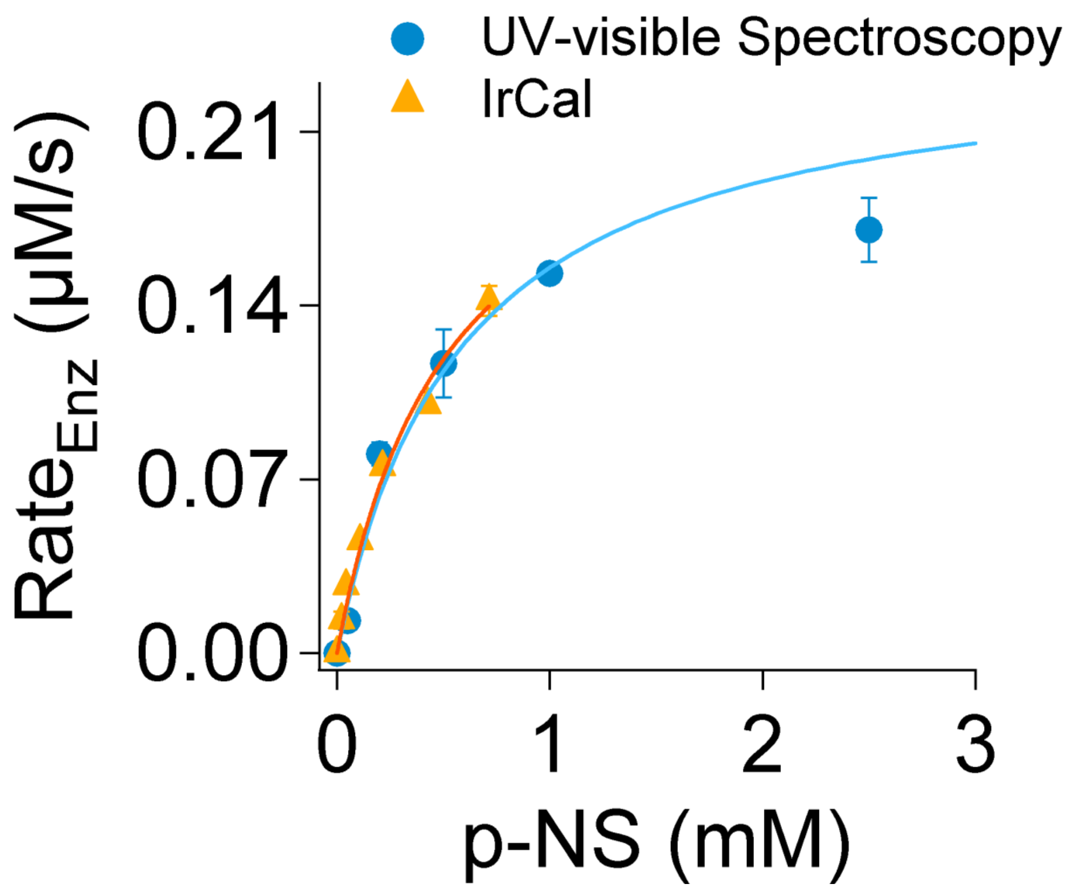

Supplementary Figure 15. The plot of the initial rate as a function of substrate concentration for conversion of p-NS by sulfatase.

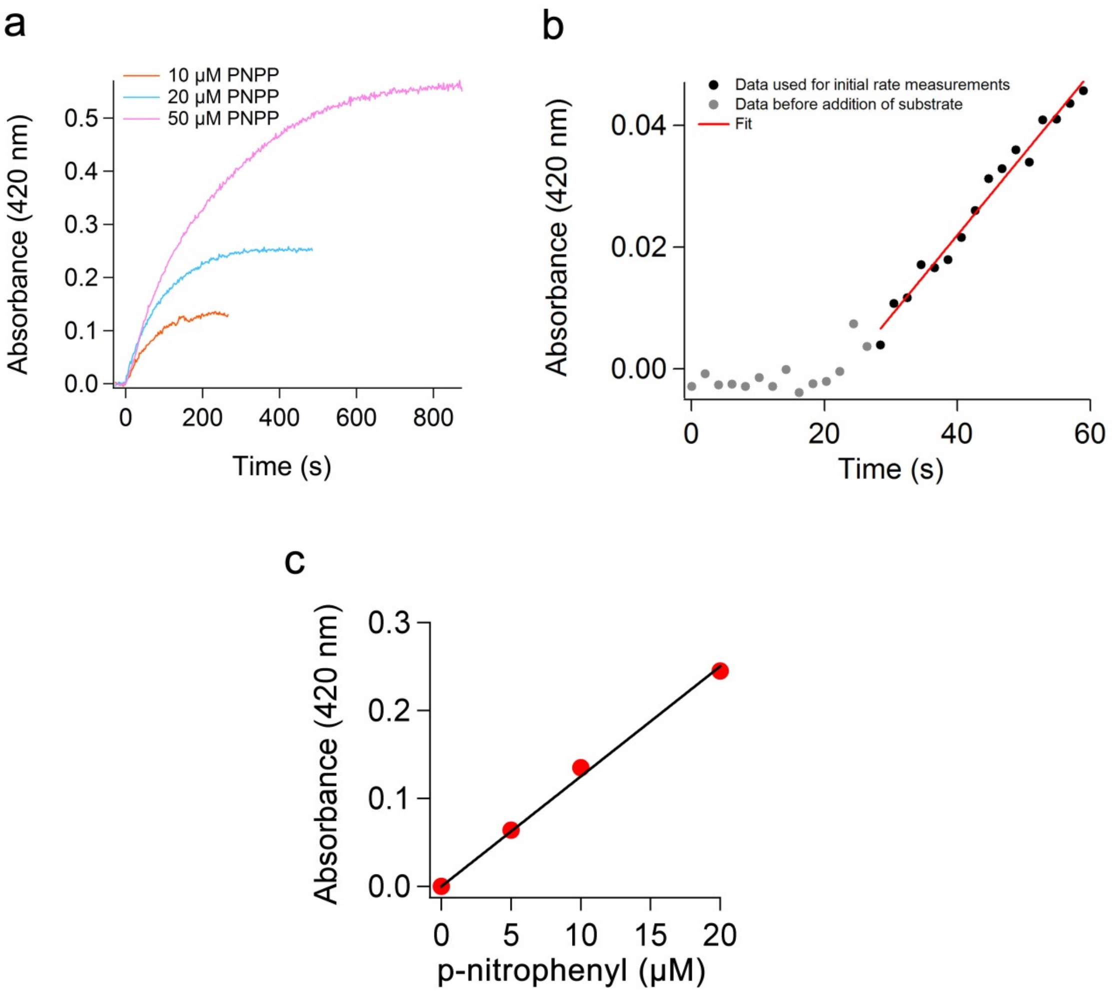

Supplementary Figure 16. (a) UV-visible spectroscopy progress curves of conversion of different amounts of p-nitrophenyl phosphate (PNPP) by alkaline phosphatase. Different amounts of substrate were added to 1 ml (final volume) of reaction solution containing 0.005 µM alkaline phosphatase. Measurements were performed at 30 °C and buffer was 100 mM Tris, 10 mM NaCl pH 8.0. Progress curves were recorded at 420 nm for formation of the product. (b) 30 seconds of data after addition of substrate were used to calculated the initial rate by fitting a line to these data points and using the slope of the line. The graph shows the measurement for final concentration of 10µM PNPP (orange line) in (a). (c) Extinction coefficient of p-nitrophenyl in 100 mM Tris, 10 mM NaCl pH 8.0 was determined at 30 °C

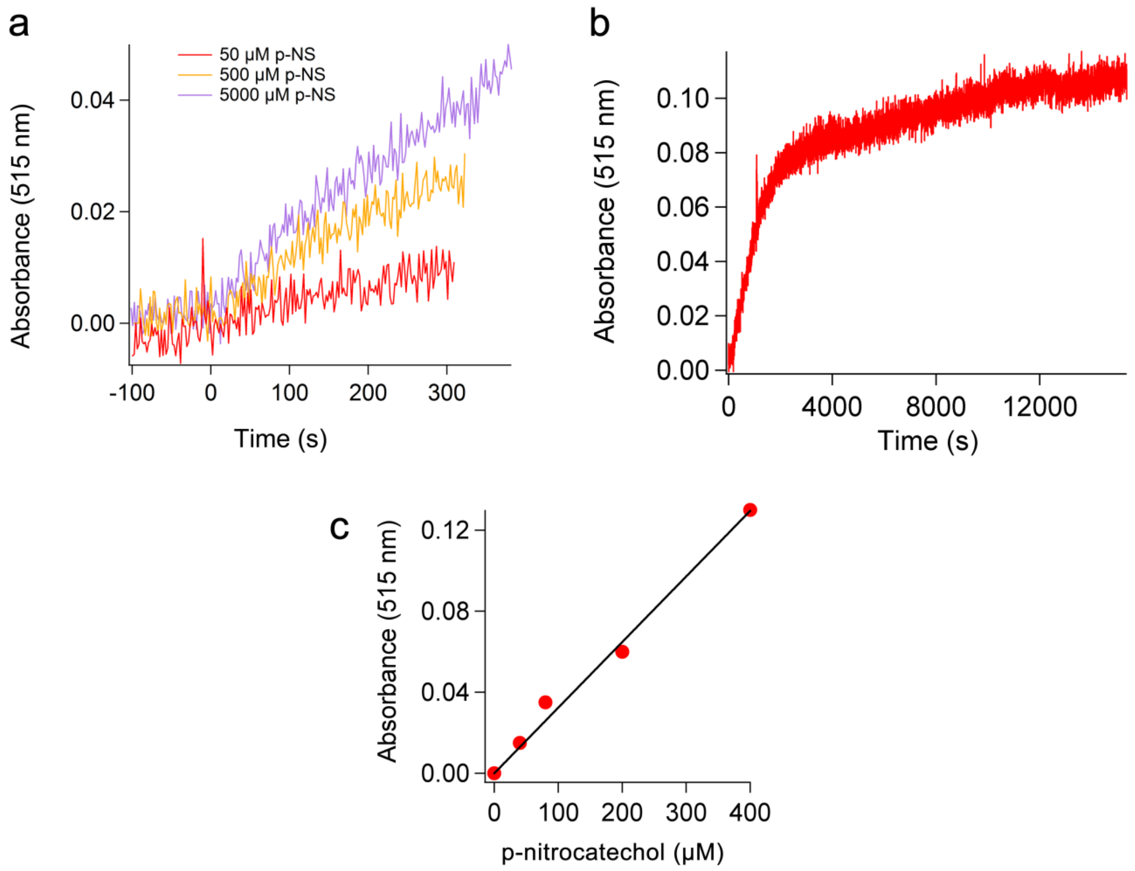

Supplementary Figure 17. UV-visible spectroscopy progress curves of conversion of p-nitrocatechol sulphate by sulfatase. (a) Different amounts of substrate were added to 1 ml (final volume) of reaction solution containing 1.75 µM sulfatase. Measurements were performed at 45 °C and buffer was 200 mM sodium acetate, 20 mM NaCl pH 5.0. Progress curves were recorded at 515 nm for formation of the product. (b) Progress curve for formation of p-nitrocatechol by sulfatase. 10 µl of 40 mM p-NS was added to final volume of 1 ml (final concentration 400 µM) and the progress curve was recorded for formation of p-nitrocatechol at 515 nm at 45 °C. To prevent the effect of evaporation, the cuvette was tightly closed. (c) Extinction coefficient of p-NS in sodium acetate buffer, pH 5.0 was determined at 45 °C.

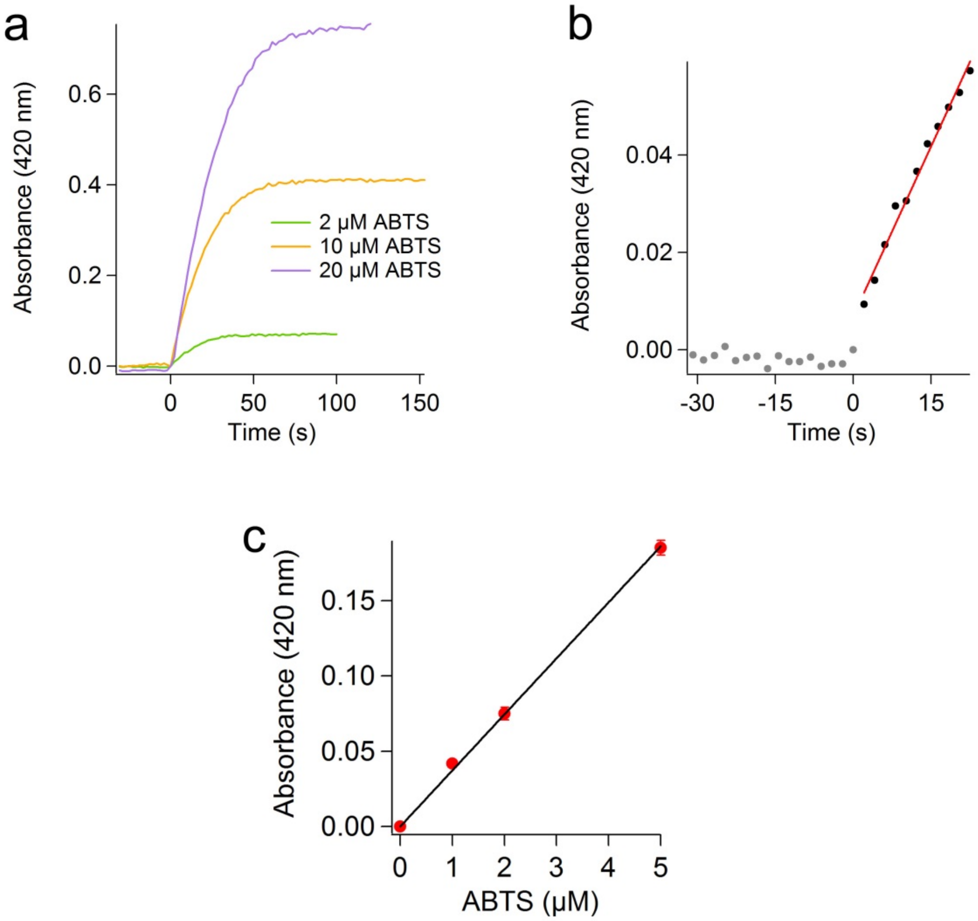

Supplementary Figure 18. (a) UV-visible spectroscopy progress curves of conversion of different amounts of ABTS by laccase. Different amounts of substrate were added to 1 ml (final volume) of reaction solution containing 0.06 µM laccase. Measurements were performed at 34°C and buffer was 100 mM phosphate, 10 mM NaCl pH 5.8. Progress curves were recorded at 420 nm for formation of the oxidized ABTS. (b) 20 seconds of data after addition of substrate were used to calculated the initial rate by fitting a line to these data points and using the slope of the line. The graph shows the measurement for final concentration of 2 µM ABTS (green line) in (a). (c) Extinction coefficient of oxidized ABTS in 100 mM phosphate buffer, 10 mM NaCl pH 5.8 was determined at 34 °C

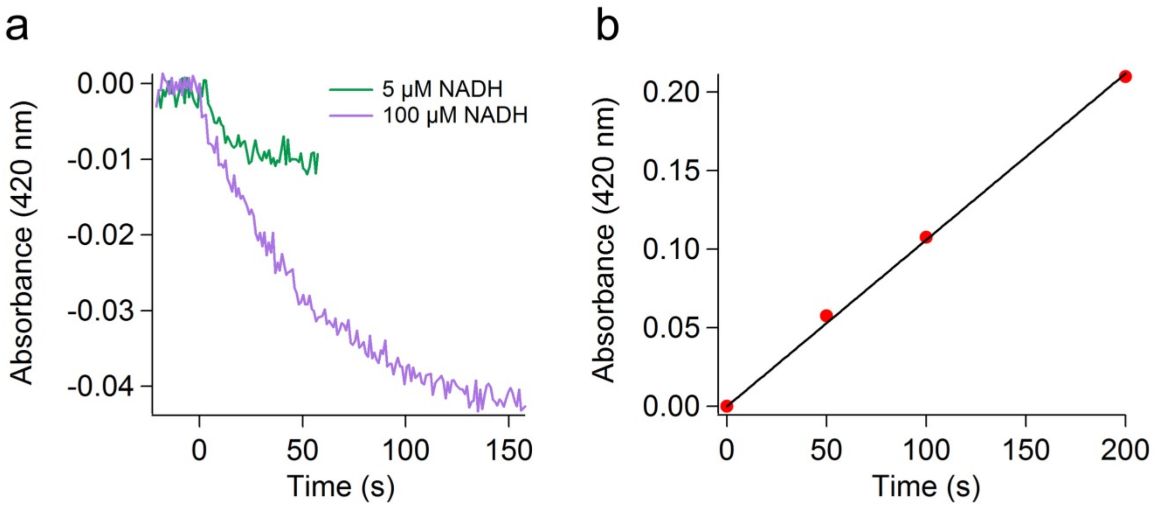

Supplementary Figure 19. (a) UV-visible spectroscopy progress curves of reduction of ferricyanide measured at 420 nm by diaphorase activity in the presence of different amounts of NADH. Different amounts of substrate were added to 1 ml (final volume) of reaction solution containing 0.082 µM diaphorase. Concentration of ferricyanide was 50 µM. Measurements were performed at 25°C and buffer was 100 mM Mops, 10 mM NaCl pH 7.0. Progress curves were recorded at 420 nm for reduction of ferricyanide to ferrocyanide. (b) Extinction coefficient of ferricyanide in 100 mM Mops buffer, 10 mM NaCl pH 7.0 was determined at 25 °C

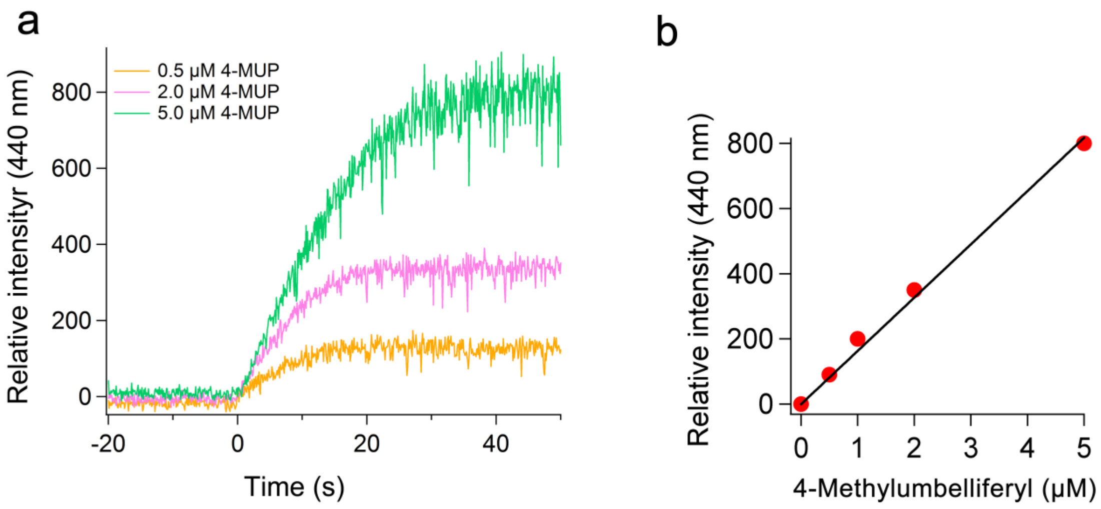

Supplementary Figure 17. Fluorescence spectroscopy progress curves of conversion of different amounts of 4-Methylumbelliferyl phosphate (4-MUP) by alkaline phosphatase. (a) Different amounts of substrate were added to 1 ml (final volume) of reaction solution containing 0.01 µM alkaline phosphatase. Measurements were performed at 22 °C, and buffer was 100 mM Mops, 20 mM NaCl pH 7.0. Excitation was performed at 360 nm and emission was recorded at 440 nm for formation of the product. (b) Extinction coefficient of 4-methylumbelliferyl product was determined in 100 mM Mops, 20 mM NaCl pH 7.0.

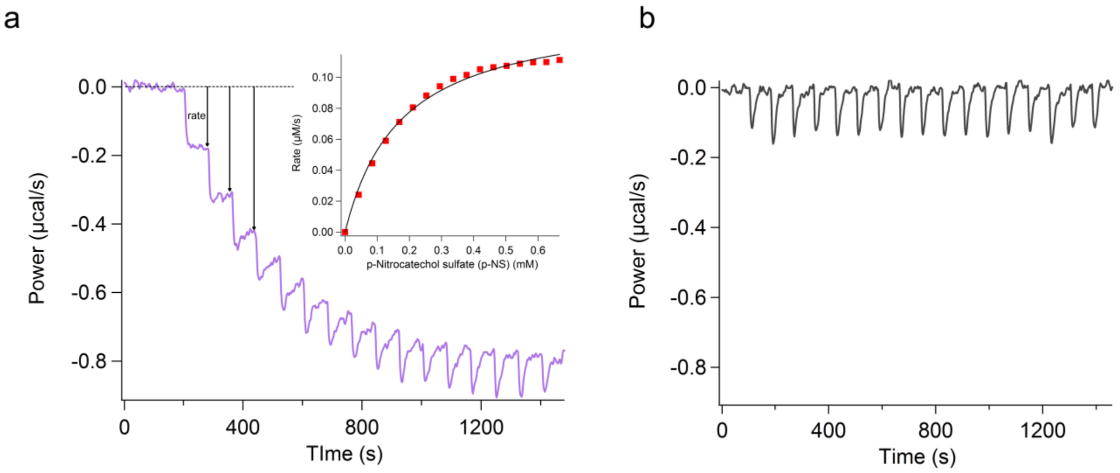

Supplementary Figure 21. Measurements of enzyme kinetic parameters using the previously proposed baseline shift (BsITC) method. (a) Multiple injection of p-nitrocatechol sulphate into the ITC sample cell that contained 0.175 µM sulfatase. The conditions were exactly the same as those used for IrCal and UV-visible spectroscopy except that the instrument was set on high feedback mode: measurements were performed at 45 °C and buffer was 200 mM sodium acetate, 20 mM NaCl pH 5.0. The inset shows the calculated rate as a function of substrate concentration using a program provided by the manufacturer based on the method proposed by Gomez et al.[1] (b) A control experiment, multiple injection of p-NS into buffer in the absence of sulfatase, was performed to check possible background reactions. No background reaction was observed. The reference power was 15 µcal/s.

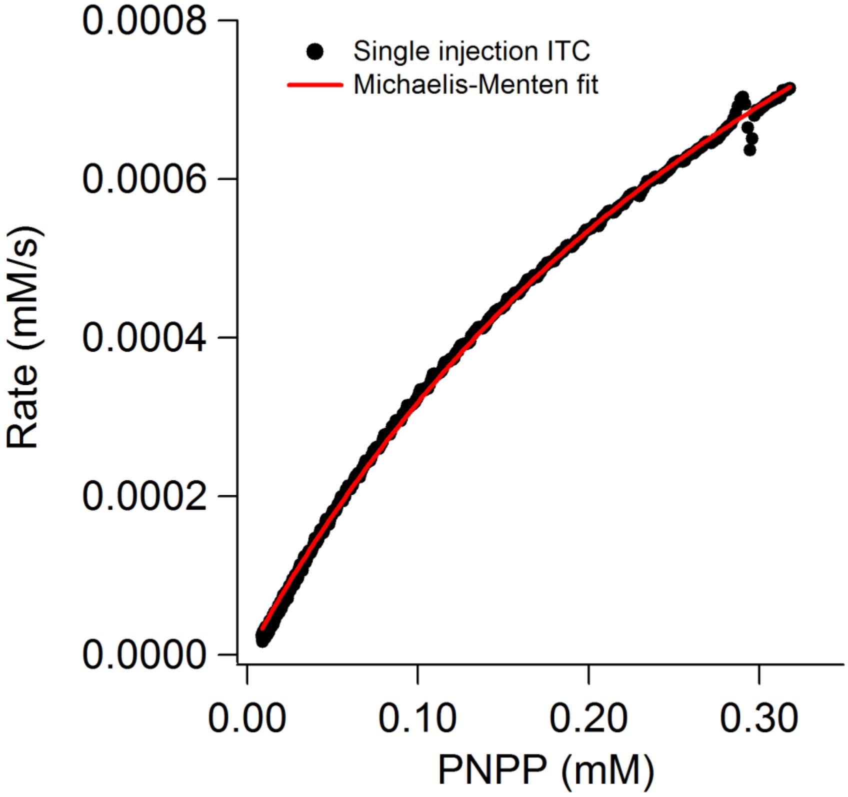

Supplementary Figure 22. Analysis of data for the single injection (SiITC) method. A single injection of 3 µl PNPP (250 mM) was performed. The concentration of alkaline phosphatase in the cell was 0.005 µM. Raw data were analyzed using the MicroCal protocol and program provided with Origine software, which are based on the method of Gomez et al.(2001)[1]. The reference power was 12.1 µcal/s.

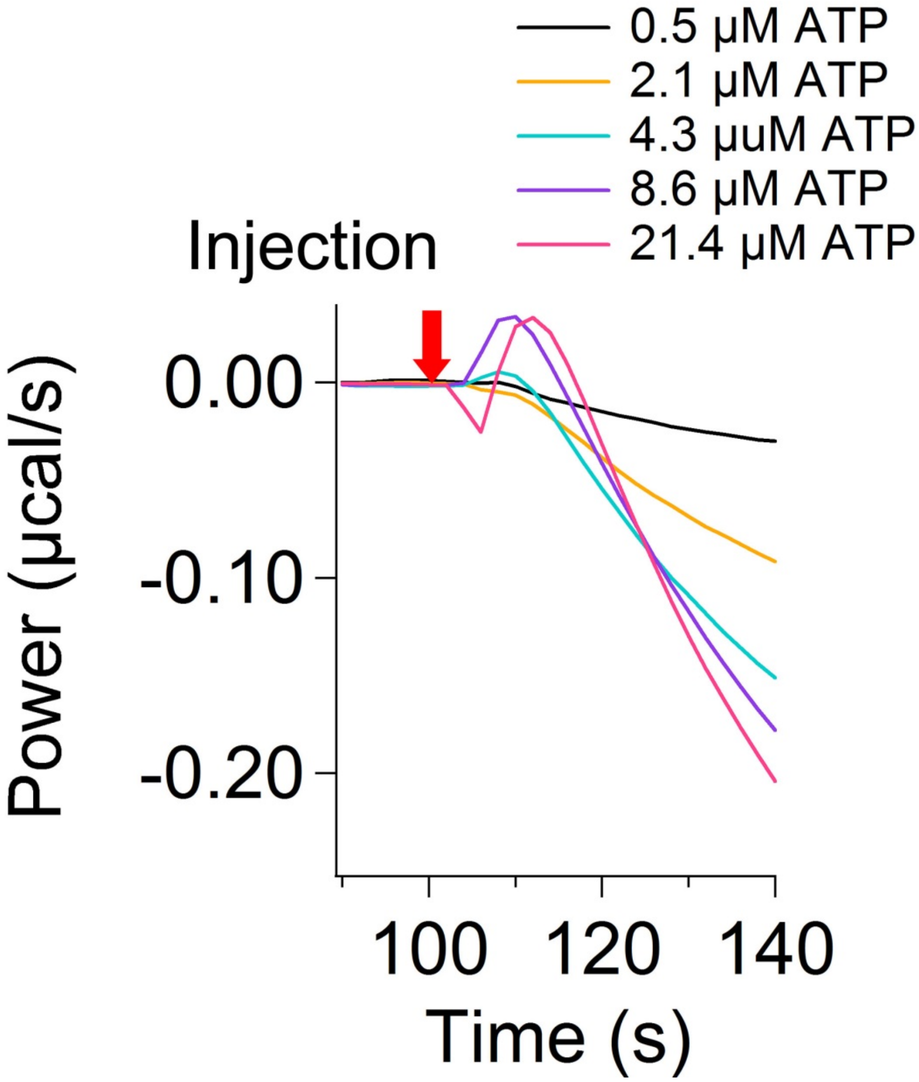

Supplementary Figure 23. The recorded ITC curves for conversion of different amounts of ATP by alkaline phosphatase (0.0037 μM). Measurements were done at 25 °C in 100 mM Mops 100 mM NaCl, pH 7.0. Data are corrected for heat of dilution of ATP in buffer.

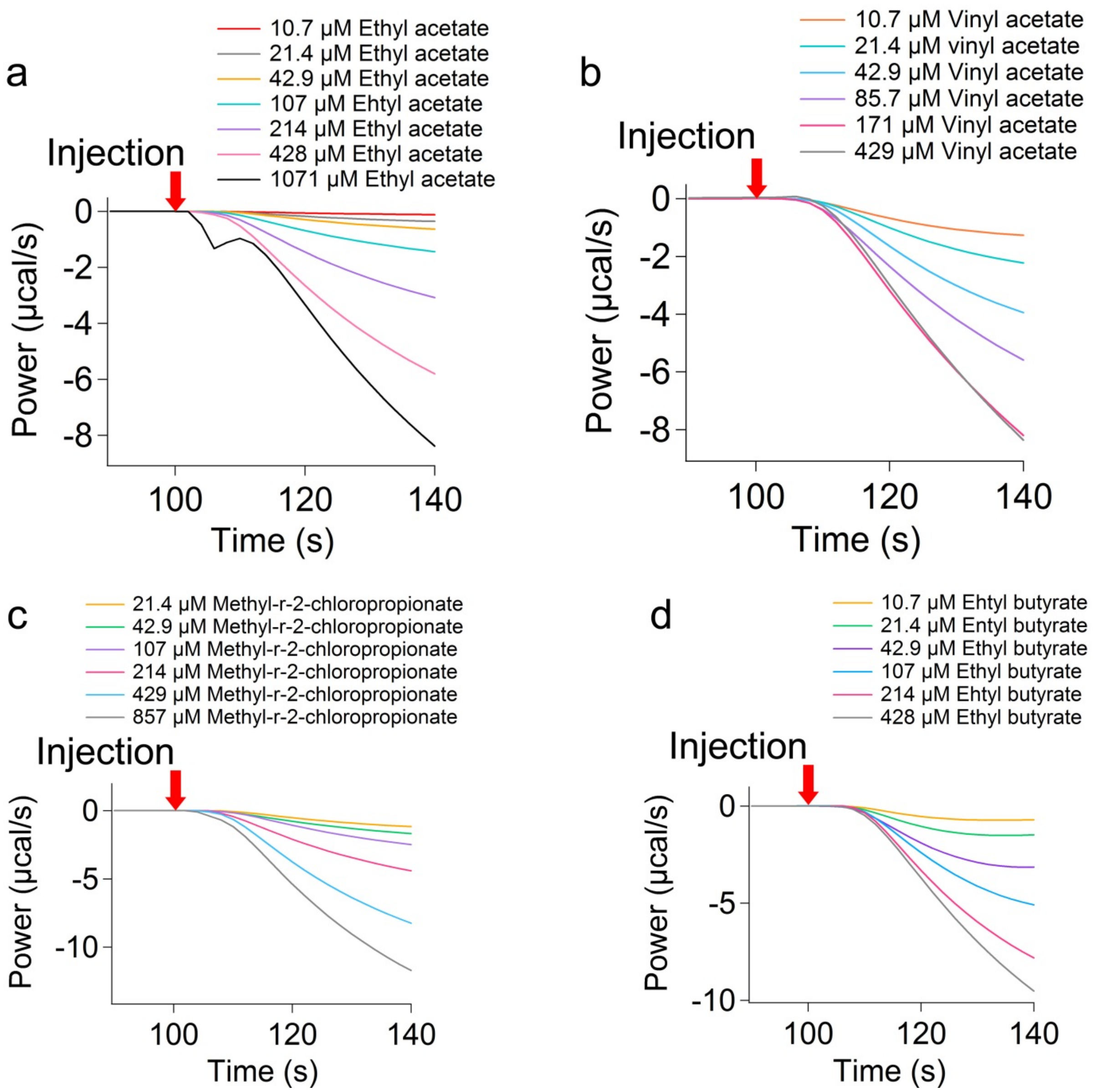

Supplementary Figure 24. The recorded ITC curves for conversion of different substrate by esterase. Conversion of different amounts of (a) ethyl acetate by 0.1 µM esterase, (b) vinyl acetate by 0.01 µM esterase, (c) methyl-r-2-chloropropionate by 0.05 µM esterase, and (d) ethyl butyrate by 0.05 µM esterase. All measurements were done at 25 °C in 100 mM Mops 100 mM NaCl, pH 7.0. Data are corrected for heat of dilution of substrate in buffer.

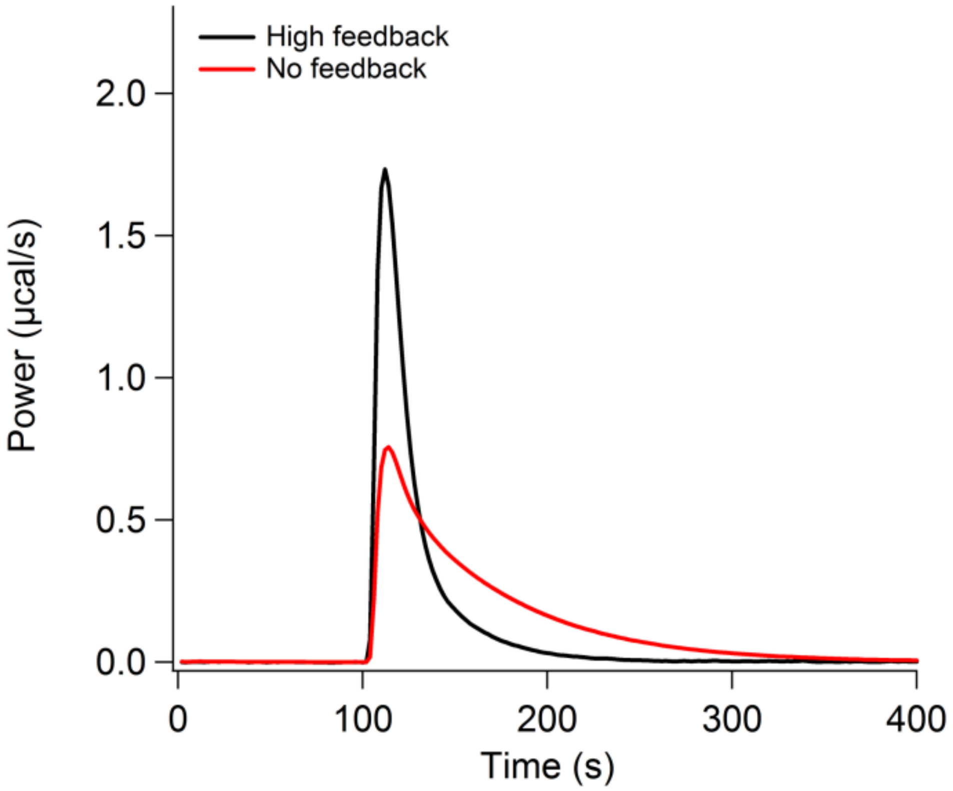

Supplementary Figure 25. High-feedback induces faster change in the power applied to the feedback heater and increases the rate of heat transfer. The endothermic process of dilution of 500 mM NaCl solution (10 mM Mops pH 7) in 50 mM NaCl solution (10 mM Mops pH 7.0) was recorded with high-feedback or no-feedback modes. 3µl of 500 mM NaCl solution was injected into the sample cell. The increase in the power of the feedback heater when the PID controller of ITC is set on high-feedback mode is greater than that when it is set on no-feedback mode. In high feedback mode the ratio of output signal (power) to the error signal (the difference between temperature of the sample cell and the reference cell) increases. As a result the power of the feedback heater increases more in a shorter time, and thus the temperature of the sample cell returns to that of the set value in a shorter time.

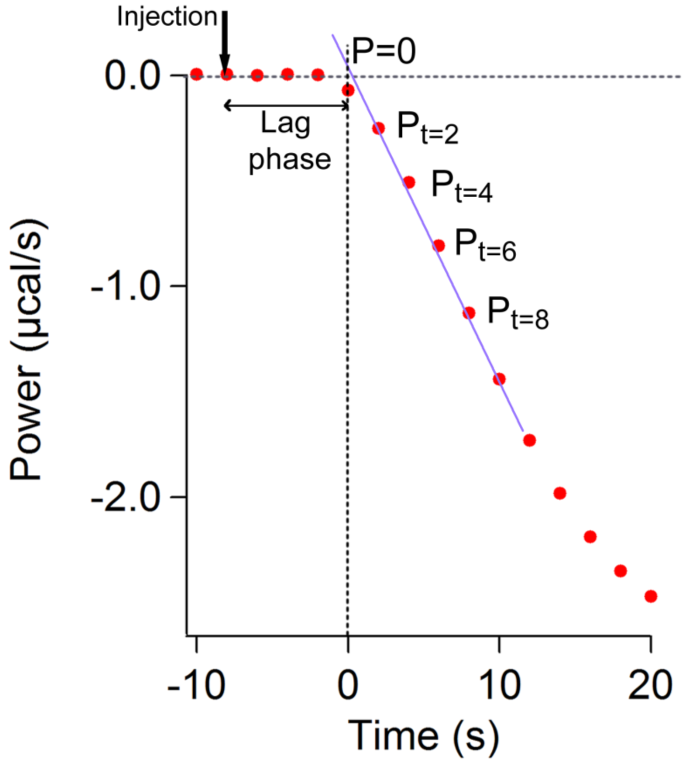

Supplementary Figure 26. Accurate determination of the lag phase. After injection of substrate under steady-state condition and full mixing a continues linear dropdown in the power is expected for an exothermic reaction. This dropdown in the power is observed after few seconds (lag phase) due to a finite mixing time and instrument related delays. Thus, the lag phase can be determined by identifying the data point that can be fitted to a linear equation. All the data points before this straight line should be removed and not used in calculation of the initial rate using our approach.

References

1 Todd, M. J. & Gomez, J. Enzyme kinetics determined using calorimetry: a general assay for enzyme activity? *Analytical biochemistry* **296**, 179-187 (2001).

2 Henzler, K., Haupt, B. & Ballauff, M. Enzymatic activity of immobilized enzyme determined by isothermal titration calorimetry. *Analytical Biochemistry* **378**, 184-189 (2008).

3 Ertan, H., Siddiqui, K. S., Muenchhoff, J., Charlton, T. & Cavicchioli, R. Kinetic and thermodynamic characterization of the functional properties of a hybrid versatile peroxidase using isothermal titration calorimetry: Insight into manganese peroxidase activation and lignin peroxidase inhibition. *Biochimie* **94**, 1221-1231 (2012).
